# A Multiphysics Biventricular Cardiac Model: Simulations With a Left-Ventricular Assist Device

**DOI:** 10.3389/fphys.2018.01259

**Published:** 2018-09-11

**Authors:** Azam Ahmad Bakir, Amr Al Abed, Michael C. Stevens, Nigel H. Lovell, Socrates Dokos

**Affiliations:** ^1^Graduate School of Biomedical Engineering, University of New South Wales, Kensington, NSW, Australia; ^2^Innovative Cardiovascular Engineering and Technology Laboratory, Critical Care Research Group, The Prince Charles Hospital, Brisbane, QLD, Australia

**Keywords:** computational modeling, multiphysics modeling, heart, LVAD, cardiac, physiome, electromechanics, fluid-structure interaction

## Abstract

Computational models have become essential in predicting medical device efficacy prior to clinical studies. To investigate the performance of a left-ventricular assist device (LVAD), a fully-coupled cardiac fluid-electromechanics finite element model was developed, incorporating electrical activation, passive and active myocardial mechanics, as well as blood hemodynamics solved simultaneously in an idealized biventricular geometry. Electrical activation was initiated using a simplified Purkinje network with one-way coupling to the surrounding myocardium. Phenomenological action potential and excitation-contraction equations were adapted to trigger myocardial contraction. Action potential propagation was formulated within a material frame to emulate gap junction-controlled propagation, such that the activation sequence was independent of myocardial deformation. Passive cardiac mechanics were governed by a transverse isotropic hyperelastic constitutive formulation. Blood velocity and pressure were determined by the incompressible Navier-Stokes formulations with a closed-loop Windkessel circuit governing the circulatory load. To investigate heart-LVAD interaction, we reduced the left ventricular (LV) contraction stress to mimic a failing heart, and inserted a LVAD cannula at the LV apex with continuous flow governing the outflow rate. A proportional controller was implemented to determine the pump motor voltage whilst maintaining pump motor speed. Following LVAD insertion, the model revealed a change in the LV pressure-volume loop shape from rectangular to triangular. At higher pump speeds, aortic ejection ceased and the LV decompressed to smaller end diastolic volumes. After multiple cycles, the LV cavity gradually collapsed along with a drop in pump motor current. The model was therefore able to predict ventricular collapse, indicating its utility for future development of control algorithms and pre-clinical testing of LVADs to avoid LV collapse in recipients.

## 1. Introduction

The optimal function of the heart relies on multiphysics phenomena, comprised of electrical activation, as well as myocardial contraction and blood hemodynamics. A deficiency in any of these physics will impact the others. As such, development of cardiac medical devices has to consider these multiphysics interactions during both pre-clinical and clinical studies. Multiphysics cardiac computational models can be a test-bed for early ideas to assess how therapeutic interventions could impact the diseased heart. This is especially beneficial to reduce the risk to patients with rare medical conditions, where the number of subjects that can be recruited for device clinical trials is limited. Results from computational simulations could be used to generate more plausible hypotheses for future testing; thus, helping to reduce development and technology transfer costs and potential clinical adverse reactions.

Numerous studies have modeled the electromechanical interactions in the myocardium with varying levels of complexity (Usyk et al., 2002; Gurev et al., 2011; Quarteroni et al., 2017). Most were coupled to Windkessel-type models to mimic the systemic circulation (Usyk et al., 2002; Gurev et al., 2011). Instead of modeling the spatially varying fluid dynamics, a uniform endocardial pressure load was calculated to constrain the volume during the isovolumic phases. Whilst such an approach may be sufficient to model most cardiac electromechanics phenomena, it limits model usability: for example, to simulate cardiac interaction with intra-cavity implants. In cases with large intraventricular pressure gradients, such as in hypertrophic obstructive cardiomyopathy (Geske et al., 2011), fluid loading on the endocardium may be spatially heterogeneous. This could render the spatially uniform pressure load constraint inaccurate. Furthermore, formation of vortices, a potential measure of cardiac health index (Hong et al., 2008), cannot be simulated in such models.

Fluid-structure interaction (FSI) models of the ventricles are important in studying fluid loading on the myocardium as well as the heart's blood pumping efficiency. For the sake of simplicity, the contribution of electrical activation on wall mechanics was not considered in many ventricular FSI models. Instead, a temporal activation function was used to govern contraction uniformly across the entire left ventricle (LV) (Nordsletten et al., 2011). Therefore, the effect of spatially heterogeneous activation on fluid behavior cannot be investigated in such models. Several studies have performed simulations of cardiac electrophysiology coupled with FSI (Watanabe et al., 2004; Choi et al., 2015). Nevertheless, their approach only utilized one-way coupling for some of the modeled physics, limiting the scope of physics interactions. A fully coupled fluid-electromechanics model can provide added advantages in terms of predictive power and future-expansion capability to test devices and drugs, or to be applied as a surgery planning tool to predict the heart's motion and blood flow profile post-surgery. The review by Quarteroni et al. (2017) provides a detailed summary of various approaches of modeling cardiac electromechanics as well as fluid-structure interactions.

A potential application of cardiac multiphysics models is to study heart-implant interactions such as the left ventricular assist device (LVAD), classified as a Class III device by the United States' Food and Drug Administration (FDA). As it may pose a high risk to patients, the device needs to be rigorously tested prior to marketing approval. Whilst the LVAD works well for patients with the most severe heart disease, excessively high pump flow rate relative to ventricular filling rate can cause the ventricles to collapse. This phenomenon is known as ventricular suction and it represents a common complication (Vollkron et al., 2007). Moreover, increased arrhythmic tendency has been noted among LVAD recipients, which may reduce right ventricular (RV) output resulting in reduced LVAD preload, worsening the suction (Robertson et al., 2017). Furthermore, several LVAD recipients also suffer from post-implantation RV dysfunction and commonly require an additional RV assist device (Neyer et al., 2016). Positioning and design of the LVAD cannula can also affect its performance (Ong et al., 2013). Therefore, LVAD complications are inherently multiphysics in nature and not only fluid-structure interactions, but also ventricular electromechanics as well as adjacent ventricular function. If any of these issues can be predicted beforehand, a strategy can be developed to lessen the risk.

In this study, we develop a multiphysics modeling framework for simulating biventricular electrophysiological behavior along with myocardial mechanics and two-way blood-myocardium interaction. The framework is implemented in an idealized biventricular structure that embeds a Purkinje fiber network to trigger myocardial electrical activation. We present computational simulations of a healthy heart as well as LVAD intervention in a dilated heart, including the impact of pump speed on both the pump and ventricles, as well as LV collapse in mitral stenosis case. We utilized commercial computational modeling software for easy replication and future model expansion.

## 2. Methods

**Figure 1:**
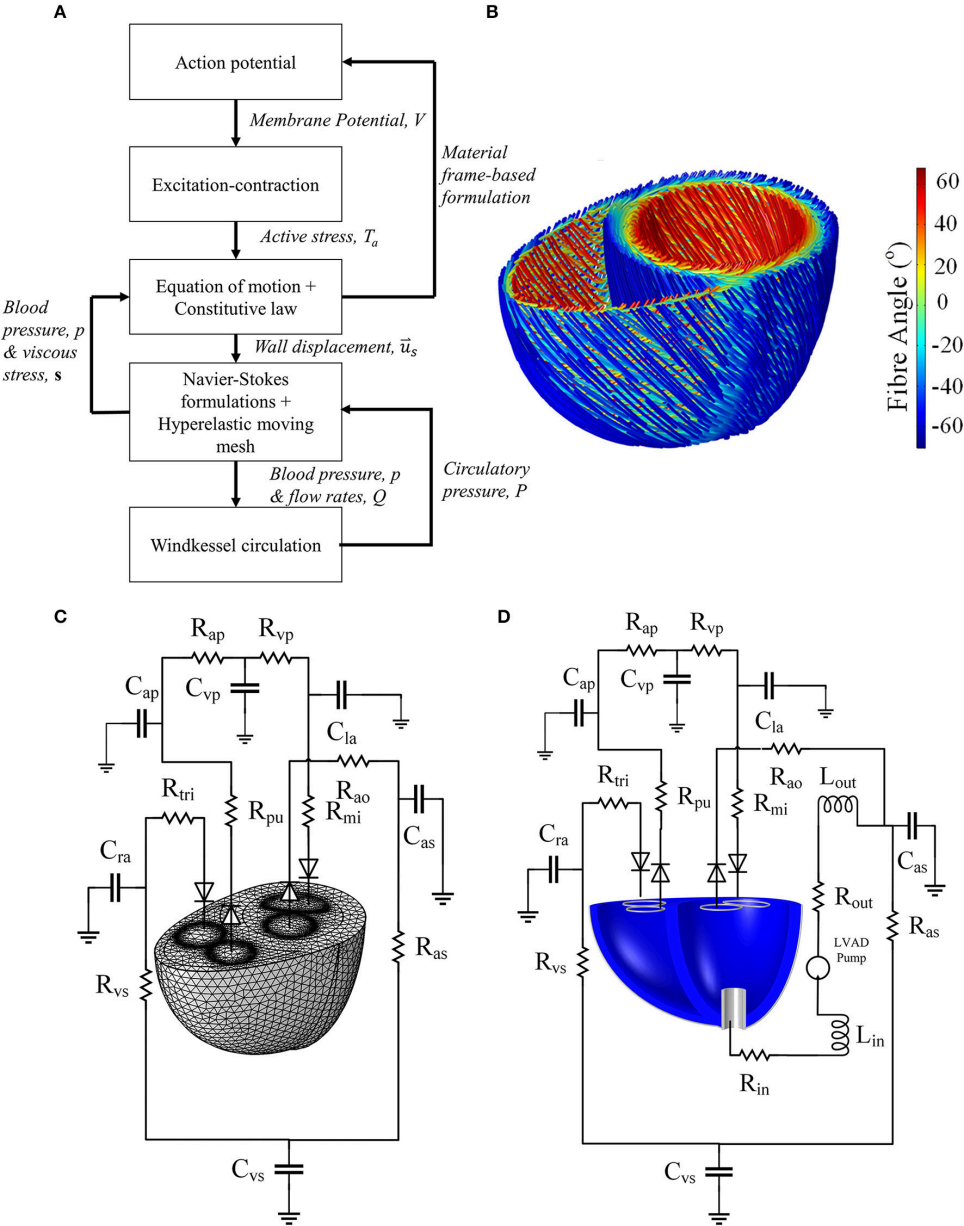
**(A)** Schematic summarizing the physics modeled, as well as the interphysics couplings. **(B)** Fiber direction streamlines for the standard model geometry of Figures 2A,B. It should be noted that the fibers are not implemented as geometrical structures but a numerical representation of the anisotropic direction of material properties. **(C)** The Windkessel circulation coupled with the normal mesh of the standard biventricular model, and **(D)** Windkessel modification for the LVAD model as well as the cannula structure implanted in the dilated heart geometry.

We first present our standard framework for formulating a healthy biventricular model based on our preliminary work presented in Bakir et al. (2017). Figure 1A summarizes all physics included along with their coupling. We then describe the implementation of heart-LVAD interaction by introducing a failing dilated heart and adding a LVAD model to the standard framework. Unless otherwise stated, descriptions and parameter values are listed in the Supplementary Material.

### 2.1. Standard biventricular model formulations

#### 2.1.1. Gross geometry and microstructure

**Figure 2:**
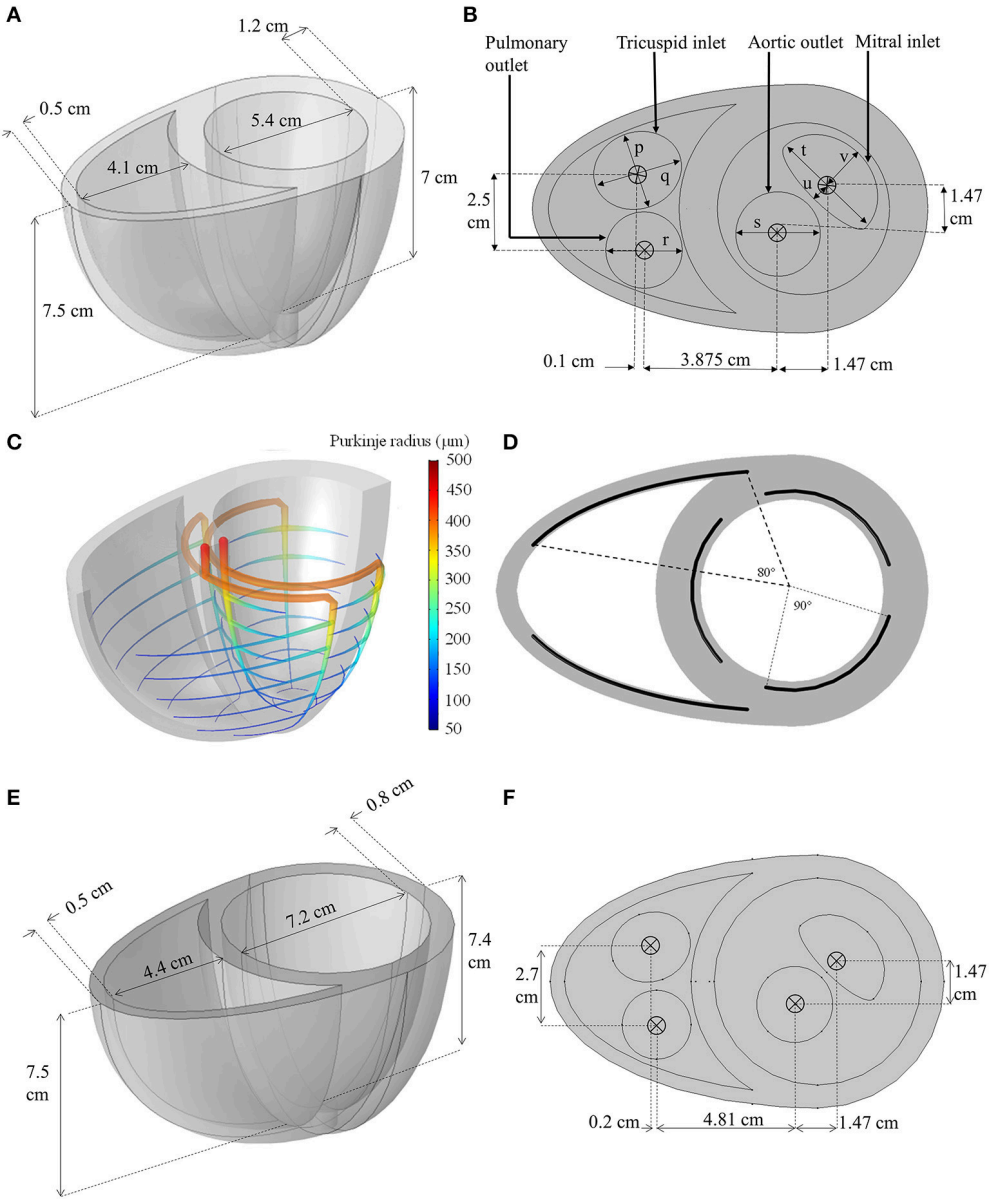
**(A,B)** Dimensions of the idealized biventricular geometry of the standard model. (*p* = 2.4 cm, *q* = 2.8 cm, *r* = 2.4 cm, *s* = 2.65 cm, *t* = 3.6 cm, *u* = 0.5 cm, *v* = 1.5 cm) **(C)** The Purkinje fiber network illustrating the change in fiber radius. **(D)** The angle span of each Purkinje-myocyte branch. **(E,F)** The dimension of the dilated heart used for LVAD modeling.

The geometry was constructed using two truncated ellipsoids merged at the interventricular sulci, as shown in Figure 2A. The aortic and mitral valve boundaries were generated using circular and semilunar shapes, respectively, whilst the pulmonary and tricuspid valve boundaries were created using circular and elliptical shapes, respectively.

To obtain a realistic activation sequence, an idealized Purkinje fiber tree structure (Figures 2C,D) was constructed within the myocardium to mimic the gross anatomy of the Purkinje network (Vigmond and Stuyvers, 2015) without restricting meshing requirements. The Purkinje structure begins at the septal base with two major bundles: the left and right bundle branches. The left bundle branch was further divided into three minor bundles near the basal septal entry, whilst the right bundle branched into anterior and posterior minor bundles. These minor bundles further branched out circumferentially, every 1 cm between the apex and base, forming the Purkinje-myocyte junctions (PMJ). The Purkinje fibers were represented in the geometry as 1D edges embedded in 3D space: and were numerically assigned a radius, *r*, that was set to decrease linearly from 500 μm at the basal origin to 50 μm at the endpoints of the PMJs based on the measurements by James (1961).

The myocardial microstructure is governed by the fiber, sheet and normal-to-sheet orientations (LeGrice et al., 1997). For simplicity, the following settings and assumptions were implemented:

1. The fiber was assumed to be −60° with respect to the circumferential plane at the epicardium, and 60° at the endocardium (LeGrice et al., 1997). The fiber angle changed linearly transmurally as shown in Figure 1.
2. The microstructural sheets were assumed to lie perpendicular to the epicardial and endocardial surfaces.
3. As the apical microstructure is difficult to measure experimentally (LeGrice et al., 1997), and adaptation of above principles could result in a singularity, the microstructure of the apex region, defined by a 1 cm diameter cylinder, was assumed to be isotropic (Nordsletten et al., 2011).

#### 2.1.2. Electrophysiology and gap junction-based propagation

Myocardial action potential (AP) formulations Equations (1)–(5) were based on the phenomenological Nash and Panfilov (2004) model, modified to incorporate units into the originally dimensionless form. Variables *V*_*m*_ and *V*_*p*_ represent myocardial and Purkinje membrane potentials respectively, whilst *R* is an auxiliary recovery variable for both myocardium and Purkinje fiber. Parameter *a* in Equations (3) and (5) was chosen to vary linearly such that *a*_*epi*_ is 0.12 and *a*_*endo*_ is 0.07 to allow the myocardium to relax in the opposite direction of its activation (Glukhov et al., 2010).

(1) $$\beta_{sv}\left(C_{m}\frac{\partial V_{m}}{\partial t}+i_{ion}+i_{pr}\right)=\nabla_{X}\cdot (\;\sigma \nabla_{X}V_{m})$$

(2) $$r\left(C_{m}\frac{\partial V_{p}}{\partial t}+i_{ion}\right)=\nabla_{X}\cdot \left(\frac{r^{2}}{2\rho_{i}}\nabla_{X}V_{p}\right)$$

(3) $$\begin{array}{l} i_{ion}=k_{1}k_{2}(V_{m}-B)\left(\left[\frac{V_{m}-B}{A}\right]-a\right)(V_{m}-1)\\ \;+k_{2}R(V_{m}-B) \end{array}$$

(4) $$i_{pr}=R_{pmj}(V_{m}-V_{p})$$

(5) $$\begin{array}{l} \frac{\partial R}{\partial t}=\left(\varepsilon_{0}+\frac{\mu_{1}R}{\left[\frac{V_{m}-B}{A}\right]+\mu_{2}}\right)\\ \;\times \left(-R-k_{1}\left[\frac{V_{m}-B}{A}\right]\left(\left[\frac{V_{m}-B}{A}\right]-a-1\right)\right) \end{array}$$

Membrane potential dynamics and AP propagation within the myocardium were governed by Equation (1), and within the Purkinje fibers by Equation (2), based on the modified cable equation developed by Alqahtani et al. (2017) to take into account the effect of varying fiber radius. Equation (3) represents the transmembrane ionic current flow while Equation (5) governs AP recovery in both the myocardium and Purkinje fiber domains. For the Purkinje fibers, parameters *a* and *k*_2_ were adjusted to achieve a three-fold increase in conduction velocity and a 2-fold increase in AP upstroke velocity relative to their respective values in the myocardium (Vigmond and Stuyvers, 2015). The Purkinje current source, *i*_*pr*_, was supplied to the myocardium only at the the PMJs to trigger myocardial activation (Equation 4).

The ∇_*X*_ operator in Equations (1) and (2) is defined within the material frame (*X, Y, Z*) such that $\nabla_{X}={\left(\frac{\partial }{\partial X},\frac{\partial }{\partial Y},\frac{\partial }{\partial Z}\right)}^{T}$, corresponding to a gap junction-based constitutive formulation described in an earlier study (Bakir and Dokos, 2015). Except at the apex, the electrical conductivity tensor, **σ**, was set to exhibit an anisotropy ratio of 4:2:1 along the fiber, $\hat{F}$, sheet, Ŝ, and normal-to-sheet, $\hat{N}$ directions respectively, in accordance with Hooks et al. (2007). The conductivity tensor, **σ**, was determined from conductivities defined in the local fiber (σ_*f*_), sheet (σ_*s*_) and normal-to-sheet (σ_*n*_) directions according to:

(6) $$\sigma =\sigma_{f}(\hat{F}⊗\hat{F})+\sigma_{s}(\hat{S}⊗\hat{S})+\sigma_{n}(\hat{N}⊗\hat{N})$$

The value of σ_*f*_ was set such that the epicardial activation time matched reported *ex vivo* experimental findings in human heart (Durrer et al., 1970). The electrical conductivity of the isotropic apex was assumed to be σ_*f*_. Zero flux boundary conditions were implemented for all myocardial boundaries unless otherwise noted.

#### 2.1.3. Active stress

The simplified form of active stress proposed by Nash and Panfilov (2004), and further modified by Göktepe and Kuhl (2010), was chosen to generate mechanical contraction triggered by the AP. Active stress, *T*_*a*_, was represented by the following phenomenological ordinary differential equation (ODE):

(7) $$\frac{\partial T_{a}}{\partial t}=\epsilon (V_{m})\left(k_{Ta}\left[\frac{V_{m}-B}{A}\right]-T_{a}\right)$$

where *k*_*Ta*_ controls the maximum magnitude of *T*_*a*_. *A*, and *B* are fixed parameters introduced here to give units to the originally dimensionless formulation. The delay function, ϵ(*V*_*m*_), was given by Göktepe and Kuhl (2010) as follows:

(8) $$\epsilon (V_{m})=\epsilon_{0}+(\epsilon_{\infty }-\epsilon_{0})\exp(-\text{exp}(-\xi (V_{m}-V_{threshold})))$$

where ϵ_0_, ϵ_∞_, ξ and *V*_*threshold*_ are also fixed parameters. The active stress was set such that the myocardial membrane potential, but not the Purkinje fiber, triggers active stress generation.

#### 2.1.4. Myocardial mechanics

The myocardium was represented by the transverse isotropic myocardial hyperelastic formulation of Holzapfel and Ogden (2009), as detailed in Equations (9)–(11), which can simulate the myocardial biaxial tension response with four parameters. A nearly-incompressible constraint was enforced through the adaptation of a volumetric strain energy function, ψ_*vol*_ in Equation (12), based on the formulation of Doll and Schweizerhof (1999):

(9) $$\psi =\psi_{isotropic}+\psi_{fiber}+\psi_{vol}$$

(10) $$\psi_{isotropic}=\frac{a_{i}}{2b_{i}}\exp(b(I_{1}-3))$$

(11) $$\psi_{fiber}=\frac{a_{f}}{2b_{f}}\exp(b_{f}{\left(I_{4f}-1\right)}^{2}-1)$$

(12) $$\psi_{vol}=\frac{\kappa (J-1)ln(J)}{2}$$

*I*_1_ in Equation (10) denotes the first invariant of the isochoric elastic right Cauchy-Green tensor, **C**, whilst $I_{4f}=\hat{F}\cdot \left(\text{C}\hat{F}\right)$. *J* in Equation (12) represents the determinant of the deformation gradient tensor, **F**. In order to ensure material stability, *I*_4*f*_ was set to zero when *I*_4*f*_ < 0, assuming that myocardial fibers do not contribute significantly to passive mechanics during compression (Holzapfel and Ogden, 2009). The apical strain energy function was solely given by ψ_*isotropic*_ due to the isotropic apex assumption.

The equation of motion governing myocardial deformation is given by Equation (13):

(13) $$\rho_{s}\frac{\partial^{2}\vec{u_{s}}}{\partial t^{2}}+\alpha \rho_{s}\frac{\partial \vec{u_{s}}}{\partial t}=\nabla_{X}\cdot \;\left(FT+\beta \frac{\partial FT}{\partial t}\right)$$

where $\vec{u_{s}}$ is the myocardial displacement. Equation (13) also contains Rayleigh damping, represented by the first order time derivative of displacement and stress, to eliminate unphysiological oscillations during fluid loading. Rayleigh damping parameters were adapted from Fritz et al. (2014) such that α = 100 *s*^−1^ and β = 0.01 *s*.

In order to couple the electrical and mechanical formulations, *T*_*a*_ was added to the 2nd Piola Kirchhoff stress, **T**, along the fiber, sheet, and normal-to-sheet directions, as shown in Equation (14) such that the magnitude of active stress in the sheet and normal-to-sheet directions was 40% of its value along the fiber direction (Usyk et al., 2002). In the isotropic apex, an isotropic active stress of *T*_*a*_ was assumed.

(14) $$T=\frac{\partial \psi }{\partial E}+T_{a}(\hat{F}⊗\hat{F})+0.4\;T_{a}(\hat{S}⊗\hat{S})+0.4\;T_{a}(\hat{N}⊗\hat{N})$$

**E** in Equation (14) is the Green-Lagrange strain tensor whilst ψ is the myocardial strain-energy function.

For simplicity, the ventricular base, including the valves, was assumed to be fixed to avoid translation and rotation. The endocardial boundary was also subjected to pressure and viscous stress from the blood fluid, discussed further below. The remaining myocardial boundaries were allowed to move freely.

#### 2.1.5. Blood hemodynamics and circulation

The fluid velocity, $\vec{v_{f}}$, and pressure, *p*, in the blood cavity were determined using the incompressible Newtonian Navier-Stokes formulation (Equation 15) and the continuity equation (Equation 16).

(15) $$\rho_{f}\frac{\partial \vec{v_{f}}}{\partial t}+\;\rho_{f}(\vec{v_{f}}\cdot \nabla_{x})\vec{v_{f}}=\nabla_{x}\cdot (-pI+\mu_{f}(\nabla_{x}\vec{v_{f}}+{\left(\nabla_{x}\vec{v_{f}}\right)}^{T}))$$

(16) $$\nabla_{x}\cdot \;\vec{v_{f}}=0$$

∇_*x*_ in Equations (15) and (16) describes the spatial derivative in the spatial frame such that $\nabla_{x}={\left(\frac{\partial }{\partial x},\frac{\partial }{\partial y},\frac{\partial }{\partial z}\right)}^{T}$.

No-slip boundary conditions were applied at the ventricular base surface except at the inlets and outlets. FSI was implemented as a strongly coupled two-way framework where (1) the fluid velocity at the endocardium was set to be equal to the endocardial surface velocity (Equation 17), and (2) the fluid total stress, **s**, a sum of pressure and viscous stress, was applied along the normal of the endocardial surface (Equation 18):

(17) $$\vec{v_{f}}|{}_{{}_{endo}}=\frac{\partial \vec{u_{s}}}{\partial t}|{}_{{}_{endo}}$$

(18) $$s=\;\hat{n}|{}_{{}_{endo}}\cdot \;(-pI+\mu_{f}(\nabla_{x}\vec{v_{f}}+{\left(\nabla_{x}\;\vec{v_{f}}\right)}^{T}))$$

To couple **s** to the solid formulation in Equation (13), a transformation from the spatial frame (i.e. the deformed frame where the Navier-Stokes equations are solved) to the material frame (i.e. the reference or material frame where the solid mechanics are solved) is needed, as shown in Equation (19), where *dv* and *dV* are mesh element scale factors in the spatial and material frame respectively.

(19) $$S=s\;\cdot \;\frac{dv}{dV}$$

Here, **S** denotes the traction from the fluid applied to the endocardial wall in material frame coordinates. The scale factors simply scale the local mesh element coordinates to the spatial and material frame coordinates.

The fluid boundary deformation was governed by the myocardial structural deformation, $\vec{u_{s}}$, whilst deformation of the mesh within the fluid domain was governed by a mesh smoothing formulation (Equation 20). The hyperelastic mesh smoothing algorithm in Equation (20) was solved to determine the minimum mesh deformation energy, **ψ_mesh_**, within the blood domain, Ω_*f*_:

(20) $$\begin{array}{l} \psi_{mesh}=\frac{1}{2}\int_{\Omega_{f}}C_{1}\left(I_{1,mesh}-3\right)+C_{2}{\left(I_{1,mesh}-3\right)}^{2}\\ \;+\kappa_{mesh}{\left(J_{mesh}-1\right)}^{2}\;dV_{\Omega_{f}} \end{array}$$

The invariants, *J*_*mesh*_ and *I*_*1,mesh*_, were given by Equations (21) and (22):

(21) $$J_{mesh}=det(\nabla_{X}\text{x})$$

(22) $$I_{1,mesh}={\left(J_{mesh}\right)}^{-2/3}\text{ tr}({\left(\nabla_{X}\text{x}\right)}^{T}\nabla_{X}\text{x})$$

where *C*_1_ and κ_*mesh*_ are artificial shear and bulk moduli, both set to a value of 1, whilst $\nabla_{X}\text{x}=\frac{\partial \text{x}}{\partial \text{X}}$, where **x** represents the spatial frame coordinates and **X** the material frame coordinates. The non-linear stiffening parameter *C*_2_ was set to 0 for the healthy simulation and 100 for the LVAD simulations to cope with greater mesh deformation subsequent to cannula inclusion. Boundary conditions for Equation (20) were set to equal the displacement of the endocardial wall, $\vec{u_{s}}$, and held fixed at the ventricular base.

The finite element model was coupled to a closed-loop Windkessel circulation (Figures 1C,D), based on the Kerckhoffs et al. (2007) circuit, modified to include an unstressed volume parameter in the capacitances, and to reflect healthy human circulation. Laminar flow rate conditions were implemented at the inlet and outlet boundaries. Each flow rate was determined from the difference between the average boundary pressure, taken at each of the inlet and outlet boundary of the finite element model, and the Windkessel circulatory pressure. The flow rate variables, described in the Supplementary Material, were applied as follows: *Q*_*mi*_ was applied to the mitral inlet, *Q*_*ao*_ to the aortic outlet, *Q*_*tri*_ to the tricuspid inlet, and *Q*_*pa*_ to the pulmonary artery outlet.

These laminar inflow and outflow boundary conditions were implemented by appending a fictitious tube to each boundary, and applying:

(23) $$L_{ext}\nabla_{x,t}\cdot [-pI+\mu (\nabla_{x,t}\;\vec{v_{f}}+{\left(\nabla_{x,t}\vec{v_{f}}\right)}^{T})]=-p_{ext}\;\vec{n}$$

where ∇_*x,t*_ is the tangential derivative in spatial frame at the inlet or outlet surfaces. This equation essentially determines the velocity applied at the boundary, assuming the inlet and outlet boundaries are connected to a tube of *L*_*ext*_ external length. A constraint, *p*_*ext*_, was applied to ensure that the flow rate through the tube matched the assigned flow rate from the above. To ensure a laminar profile and prevent back-flow when the valve is closed, *L*_*ext*_ needs to be sufficiently large to eliminate numerical backflow was set to 20 m in our model. It should be noted that the *L*_*ext*_ does not represent an actual physical tube length, but a value chosen to satisfy the conditions.

#### 2.1.6. Simulation protocol

1. To begin, the Windkessel circuit was decoupled from the heart and the LV and RV chambers were inflated until they reached their end diastolic volumes, *V*_*lv*,∞_ and *V*_*rv*,∞_, respectively. The filling rate for each ventricle was calculated using Equation (24) based on a proportional controller:
  (24) $$Q_{fill}=-k_{flow}(V_{lv}-V_{lv,\infty })$$
  where *k*_*flow*_ is a fixed parameter set to 200,000 s^−1^.
2. Using the filled ventricle, the Windkessel circuit was connected and the model was solved using the initial Windkessel volumes listed in the Supplementary Material, which were tuned to be near their steady-state cycle' volumes. The model was run for three steady cycles. A pulse stimulus of 2 ms duration at 60 beats per minute (bpm) was applied at the basal entry of both left and right bundles of the Purkinje network.

### 2.2. LVAD simulations

A left ventricular assist device (LVAD) model was attached to the biventricular model via a cannula.

#### 2.2.1. Heart failure modifications

Prior to adding the LVAD, the biventricular model was modified to reflect dilated cardiomyopathy (DCM), a common etiology of LVAD recipients. The geometry was modified by increasing the LV short axis and reducing its wall thickness as shown in Figures 2E,F (Mohiaddin, 1995; Dandel et al., 2008). This increased the LV chamber size whilst the RV chamber size was minimally affected. The heart rate was increased to 80 bpm (Mohiaddin, 1995; Dandel et al., 2008) and the LV contraction strength was reduced by setting the *k*_*Ta*_ parameter of the standard model to half its value, in accordance with isometric twitch measurements in isolated epicardial tissue obtained from DCM patients (Hasenfuss et al., 1992). Other settings described in the standard model were left unchanged.

#### 2.2.2. LVAD model

The LVAD was represented by an ODE model that relates the hydraulic state experienced by the pump and pump motor current. The LVAD model, developed by Lim et al. (2010), consists of three components:

1. Motor windings electrical equation, describing the current, *I*, needed to maintain the pump motor speed, ω:
  (25) $$V_{pump}=-2k_{e}\;\omega +R_{pump}\;I+L\frac{dI}{dt}$$
  The voltage, *V*_*pump*_, was controlled using a proportional controller, which maintains the pump speed at the desired speed, ω_*set*_:
  (26) $$V_{pump}=k_{pump}\;(\omega -\omega_{set})$$
2. Electromagnetic torque transfer equation, relating the electromagnetic torque, *T*_*e*_, produced by the pump to the input flow rate, *Q* (L min^−1^):
  (27) $$T_{e}=3k_{e}\;I=J\frac{d\omega }{dt}+a_{p}\;Q^{2}\;\omega +b_{p}\;Q\;\omega^{2}+c_{p}\;\omega +d_{p}\;\omega^{3}$$
3. Pump hydraulic equation, relating the differential pressure, Δ*P*, produced by the pump to the input flow rate and pump speed:
  (28) $$\Delta P=e_{p}+f_{p}\;Q^{3}+g_{p}\;\omega^{2}$$
  Pump inflow rate was obtained via the equation:
  (29) $$\frac{dQ}{dt}=\frac{\Delta P-(P_{as}-P_{lv,cannula})-(R_{in}+R_{out})\;Q}{L_{in}+L_{out}}$$

Variable inflow and outflow resistances, *R*_*in*_ and *R*_*out*_, were set to be proportional to the pump flow rate to account for the turbulence effect in the pump (Lim et al., 2010), and are given by

(30) $$R_{in}+R_{out}=k_{r}\;Q$$

The LVAD equations were linked to the ventricular model via the numerical difference between LV pressure averaged at the cannula outlet boundary, *P*_*lv,cannula*_, and the systemic arterial pressure, *P*_*as*_ of the Windkessel circulation as shown in Equation (29). This difference is the pressure head of the pump, Δ*P*, minus the pressure loss across the cannula. Equations (25)–(29) describe the relationships that determines the overall pump flow rate, *Q*, using this pressure difference.

#### 2.2.3. Cannula design

A cylindrical generic pump cannula, 1.6 cm inner diameter and 0.2 cm wall thickness, was inserted at the LV apex of the biventricular geometry as shown in Figure 1C. The cannula extends only a quarter of the LV apicobasal distance as suggested by Ong et al. (2013). The cannula was set to be a linear elastic silicon material (*E* = 170 GPa, ν = 0.28, ρ_*s*_ = 2,329 kg m^−3^), was held fixed in space at its external parts outside the LV.

Contact modeling between the LV endocardium and the cannula is necessary to prevent mesh collapse when surface contact occurs. In essence, the contact formulation applies loads that are inversely proportional to the distance between contacting surfaces when the surfaces move closer than a certain threshold. A wall distance formulation (Fares and Schröder, 2002), described in the Supplementary Material, was applied to calculate the distance between the endocardial surface and contact destination surfaces (cannula and ventricular base). The ventricular base of the fluid domain was included as a contact destination, due to the possibility of the collapsing wall contacting the basal surface, inducing mesh collapse.

A boundary load *P*_*contact*_, given by Equation (31), was applied along the normal direction of the endocardial surface to counter its movement toward the contact destination surfaces:

(31) $$P_{contact}=\{\begin{array}{ll} k_{contact}(G_{contact}-G_{contact,lim}) & G_{contact,lim}\leq G_{contact}\\ 0 & \text{otherwise} \end{array}$$

*G*_*contact*_ is the reciprocal of distance between surfaces, whilst *G*_*contact,lim*_ is the set threshold (150 m^−1^). *k*_*contact*_ was set to 10^3^ N mm^−1^, which was sufficient to prevent the wall contact. To enhance the blood domain mesh deformation with the added cannula, parameter *C*_2_ in Equation (20) was set to 100, introducing a non-linear stiffening to the mesh smoothing formulation.

#### 2.2.4. Simulation protocol

1. The dilated heart model was run for three cycles without the LVAD cannula added.
2. The LVAD was connected to the dilated heart model at the low-end speed of 2,100 RPM and run for three stable cycles.
3. The model was run for half a cycle (systolic) at 2,100 RPM, followed by a 300 ms duration where the pump speed was linearly ramped up to 150% of its original speed (3,150 RPM). This speed was held for three cycles, and the impact of this increased speed on the heart was observed. This was performed to determine whether aortic ejection ceased at high speed, as observed by Lim et al. in their simulation and animal experiments Lim et al. (2010).
4. To simulate suction or ventricular collapse, the LV preload was reduced significantly. Starting from the fourth cycle at 3,150 RPM pump speed, mitral stenosis was induced by increasing mitral resistance, *R*_*mi*_ by 25-fold and then running the model for three more cycles.

### 2.3. Mesh settings

The mesh was constructed using a combination of tetrahedral, pyramidal and prism elements. A fluid boundary layer of two elements was placed at the fluid domain side of the fluid-structure interface. The outlet and inlet surface meshes were refined by setting their edges to an average element size of 0.1 cm. The resulting normal mesh is shown in Figure 1C. Mesh element size was determined as the length of the element's largest edge. The average mesh size for the myocardium was 0.52 cm whilst in the fluid domain it was 0.4 cm, which resulted in 22661 myocardial mesh elements and 44078 blood domain elements. A mesh convergence analysis was conducted on the standard model by reducing the average element size to 0.35 and 0.31 cm for the myocardium and fluid domains respectively, defined as the finer mesh setting from here on. The LVAD simulation was computed using a similar setting to the normal mesh to the standard healthy heart simulation, but with an average element size of 0.21 cm within the cannula. This resulted in 28005 myocardial mesh elements and 103569 blood domain elements.

Mesh convergence analysis was performed for the biventricular model using the following quantities:

1. Characteristic quantities, *C*_*q*_
  (32) $$C_{q}=\frac{1}{N}\sum_{n=1}^{N}\frac{1}{M}(\sum_{m=1}^{M}\zeta_{mn})$$
2. Relative root mean square, $\%\bar{RMS}$
  (33) $$\%\bar{RMS}=100(\frac{\sqrt{\frac{1}{M}\sum_{m=1}^{M}{\left(\zeta_{m,normalmesh}-\zeta_{m,refinedmesh}\right)}^{2}}}{\sqrt{\frac{1}{M}\sum_{m=1}^{M}{\left(\zeta_{m,refinedmesh}\right)}^{2}}})$$

where the ζ_*mn*_ quantities represent the local electrical activation time, myocardial displacement and fluid pressure at the times of peak LV ejection and peak LV filling rate at a set of 40x40x40 material datapoints generated across the biventricular structure. The magnitudes of the first derivatives of *V*_*m*_, $\vec{u_{s}}$, $\vec{v_{f}}$ and *p* were extracted, and electrical activation time was determined by finding the time of maximum first derivative of *V*_*m*_ at each point.

### 2.4. Solver settings

The physics involved in the model are summarized in Figure 1A. In brief, the myocardial AP (Equation 1) was triggered by the Purkinje current (Equation 2). AP activation triggers the generation of active stress in Equation (7). This active stress was in turn coupled to the transverse isotropic hyperelastic myocardial formulation by directly adding the active stress tensor (Equation 14) into the 2nd Piola-Kirchoff stress in Equation (14). The latter was determined from the derivative of the strain energy function with respect to the strain components, as indicated in Equation (14). The electromechanics formulations (Equations 1–14) are implemented only in the myocardial domain and solved in the material frame, to consider for gap junction-controlled AP propagation as described in Bakir and Dokos (2015).

The fluid physics (Equations 15–16) was solved in the spatial frame within a moving mesh. The fluid-solid coupling occurs at the endocardial surfaces (Equations 17–19), with the fluid velocity set to the endocardial wall velocity and fluid total stress, **S**, applied as a boundary load for the myocardial mechanics. Deformation of the mesh in the blood domain was governed by the hyperelastic formulation (Equation 20), with boundary conditions given by the endocardial displacement (Equation 13) while the basal plane was held fixed.

We utilized COMSOL Multiphysics 5.2a (COMSOL A.B., Sweden) for generating the geometry and mesh as well as solving all formulations. Fully-coupled PARDISO direct linear solver with an automatic damped Newton method were implemented, where all formulations were assembled in a single matrix and solved simultaneously together with preordering algorithm set to nested dissection multithreaded. Streamline diffusion and crosswind diffusion artificial stabilizations of the Navier-Stokes equations were applied, which enabled same-order velocity-pressure numerical integration and eased the requirement for a very fine fluid domain mesh. We adopted P1-P1 linear Lagrange elements for the Navier-Stokes equations and quadratic Lagrange elements for the membrane potential potential and recovery, *R*, variable as well as for all the solid mechanics formulations. All formulations were solved simultaneously and all variables were updated at each time step using a second order Backward Differentiation Formula (BDF) adaptive time-stepping method with an event detection algorithm implemented to ensure the BDF solver did not miss the stimulus events. Simulation results were output at 2 ms time steps.

## 3. Results

### 3.1. Standard biventricle model

#### 3.1.1. Overall multiphysics behavior

**Figure 3:**
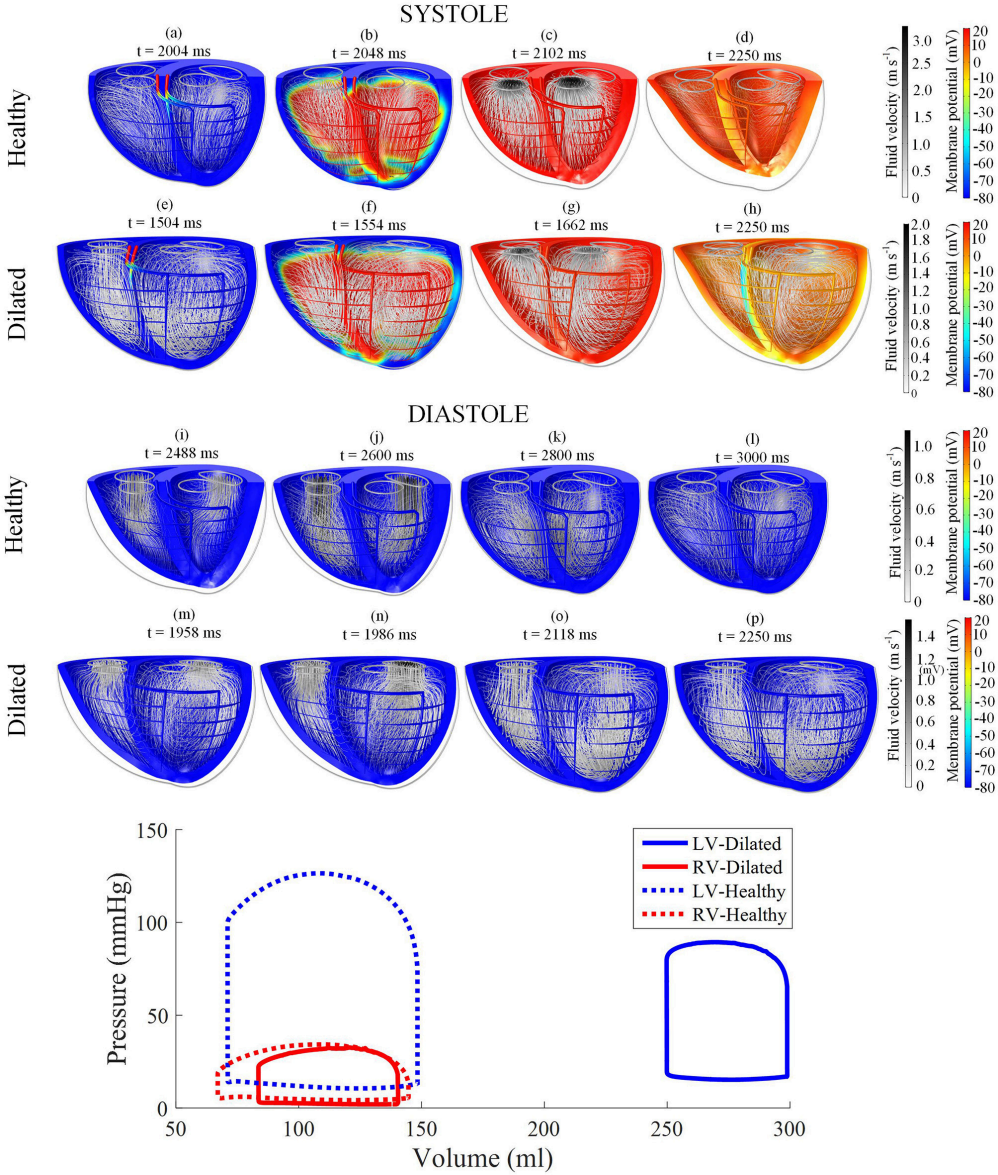
Snapshots of the biventricular multiphysics model depicting the myocardial membrane potential, myocardial movement and fluid velocity streamlines taken at various time points during systole and diastole. **(a–d,i–l)** were taken from the healthy heart model. **(e–h,m–p)** were from the dilated heart model. The stimulus was applied at *t* = 2,002 ms for the healthy model and *t* = 1,502 ms for the dilated model. The pressure-volume relations are displayed at the bottom of the figure. Streamline tube radius is proportional to the blood velocity magnitude. The animations of healthy model and dilated heart model are available in Supplementary Animations 1, 2 respectively.

Electric activation, myocardial motion, and blood velocity vector streamlines are shown in the snapshots of model behavior in Figure 3 and in the Supplementary Animation 1. The snapshots were taken at various phases in the third cycle of the steady cycle model.

The model started from the end-diastolic volume (EDV) phase, where vortices from the previous filling phase still persist in both ventricles. The stimulus began at the basal entries of the Purkinje fibers. As the myocardium began to electrically activate, a torsional motion was noted. The blood flow vortices began to diminish and the flow was gradually directed toward the outlets until ejection was initiated. As the heart reached total electrical activation, blood flow streamlines were oriented toward the outlets in near straight lines. The apex also moved toward the base during contraction. The straight streamlines gradually diminished as the heart entered isovolumic relaxation.

AP relaxation first occurred in the epicardium, whilst the endocardium repolarized last: the opposite of the activation direction. The filling was initiated even before the heart contraction fully relaxed. As the ventricles were being filled, blood vortices formed near the inlets. The vortices grew larger and moved apically. LV vortex shape was somewhat symmetrical whilst the RV displayed a more elongated and crescent-like shape due to the RV geometry. At the end of the cycle, the ventricles returned back to their starting EDV shape with larger vortex presents within the central region of the ventricular cavities. Smaller vortices can also be noted in the lateral regions nearing the myocardial wall, near the inlets.

#### 3.1.2. Electrical activation

Following external stimulation of the basal entries of the Purkinje network, the AP propagated through the branching fibers toward the apex. The Purkinje fiber network took 34 ms to be wholly activated. As the AP traveled through the PMJ edges the myocardium was excited. The site of first myocardial activation occurred at the septal region 20 ms after the Purkinje base was stimulated.

**Figure 4:**
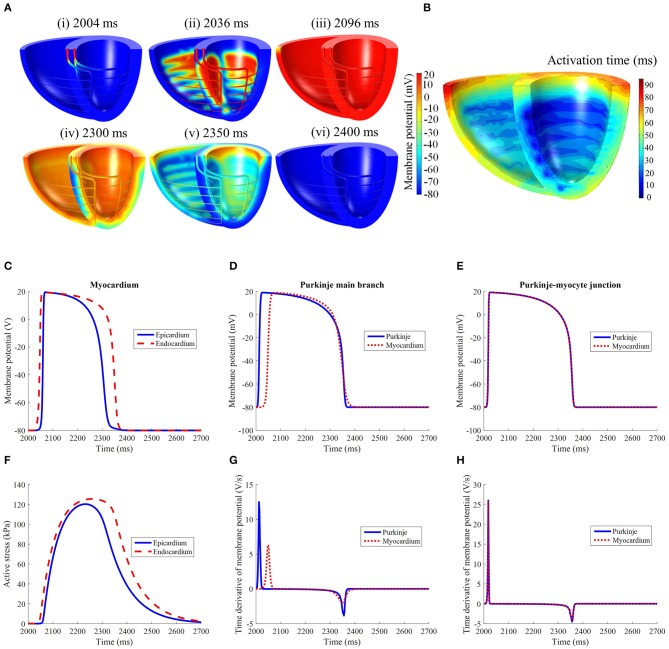
**(A)** Electrical activation in the standard model following initiation at *t* = 2,002 ms. It should be noted that the recovery spreads in the opposite direction of activation. **(B)** Electrical activation time map in the standard model. Comparison of **(C)** LV free wall myocardium endocardial and epicardial, **(D)** Purkinje fiber, and **(E)** Purkinje-myocyte junction action potential profiles. **(F)** Active stress profile of the myocardium taken at the same location as **(C)**. The time derivative of membrane potential of the **(G)** Purkinje fiber and **(H)** Purkinje-myocyte junction, taken at the same locations as in **(D,E)**.

LV activation was initiated at three endocardial sites that merged into a single wavefront, which propagated radially toward the epicardium before finally reaching the base (Figures 4A,B). Epicardial breakthrough occurred first in the anterior and posterior interventricular sulci. These breakthroughs formed two wavefronts that propagated toward the unactivated regions in the RV. These wavefronts moved in a near tangential fashion at the free wall of the RV toward the base. Overall, it took 94 ms for the whole structure to be activated, with the RV free wall base the last activated region. On the other hand, the repolarization sequence was the opposite of the activation sequence such that it was initiated at the epicardium rather than the endocardium.

The action potential and active stress profiles are displayed in Figures 4C–H. The APD90 measured at the endocardium and epicardium was 324 and 266 ms, respectively. The presence of this APD gradient was sufficient to generate a recovery sequence opposite to that of the activation sequence. The Purkinje AP morphology exhibited an upstroke velocity twice that of the myocardium and the APD was longer (Figure 4D). At the Purkinje-myocyte junction (PMJ), the myocardial AP appeared similar to the Purkinje AP morphology. The time to peak active stress was 192 and 222 ms whilst the time to half relaxation was 142 and 156 ms in the epicardium and endocardium, respectively. These measures were extracted from three material points, located mid-apicobasal distance of the LV free wall, septum and RV free wall.

#### 3.1.3. Mechanical variables

The fiber, sheet, and normal-to-sheet components of the 2nd Piola-Kirchoff stress and Green-Lagrange strain tensors were extracted from three material points, located at the epicardium of the LV free wall, septum and RV free wall. These points were taken at midway between the base and apex.

**Figure 5:**
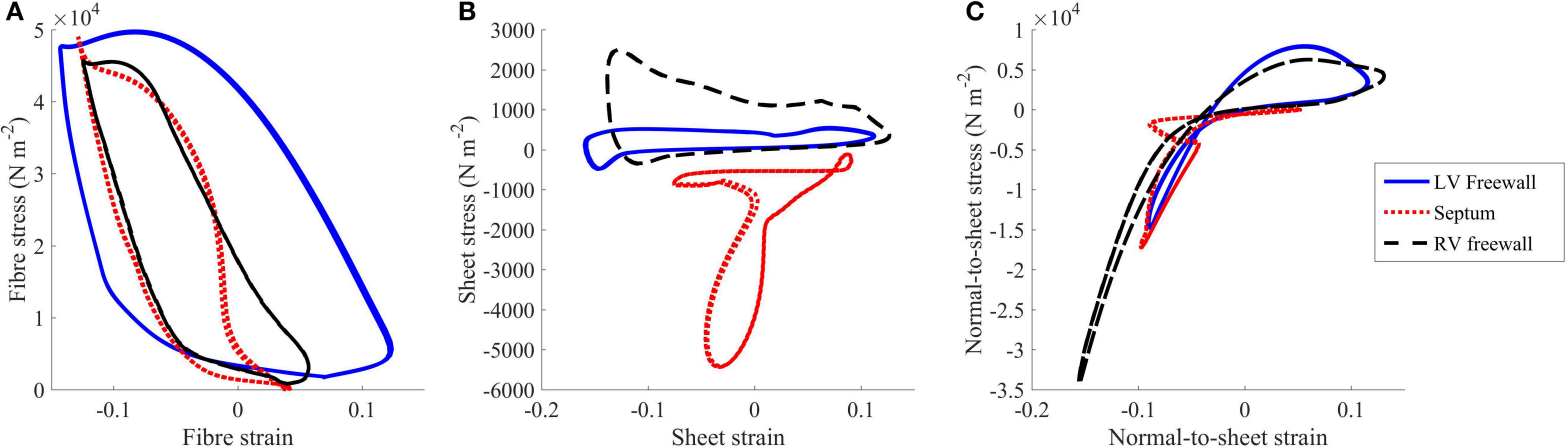
Stress vs. strain plots taken from the **(A)** fiber, **(B)** sheet, and **(C)** normal-to-sheet components of the 2nd Piola-Kirchoff stress and Green-Lagrange strain tensors. The results are taken from the standard healthy model at the third cycle (*t* = 2,000–3,000 ms).

To assess regional work, stress-strain loops were generated (Figure 5) as in Russell et al. (2012). The fiber direction was the dominant direction of deformation and stress. The LV free wall stress-strain loop was the largest compared to the those measured at the septum or RV free wall, indicating greater regional work in the LV free wall relative to the other regions. The stress strain loops for the sheet and normal-to-sheet components showed more skewed and distorted loops.

**Figure 6:**
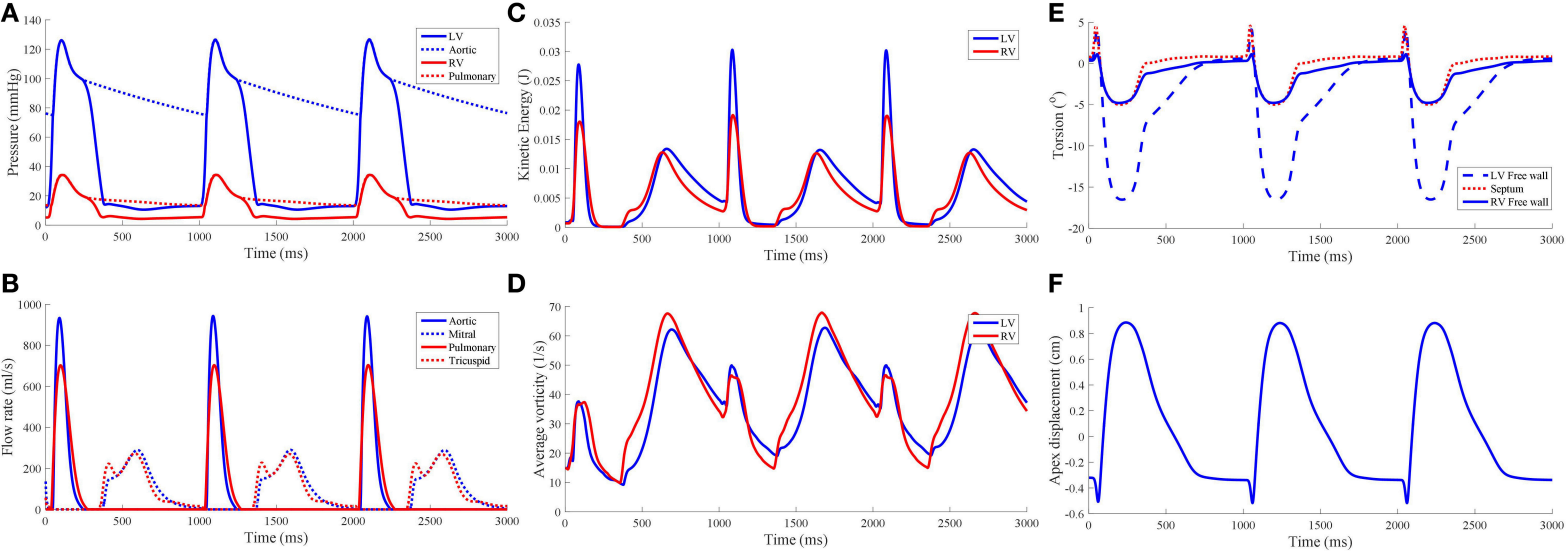
Hemodynamic measures from the standard model, namely **(A)** pressure waveforms, **(B)** inlet and outlet flow rates, **(C)** blood kinetic energy, and **(D)** average vorticity. Global mechanical measures of ventricular torsion and apex displacement are displayed in **(E,F)**, respectively.

Torsional motion was assessed at three points in the myocardium: LV free wall, septum and RV free wall. These material points were positioned near the apical region at three quarters of the apicobasal distance. All points were placed at the epicardial surfaces. Torsion was mostly predominant in the LV free wall, with 16.5° counterclockwise motion when viewed upward from the apex, as shown in Figure 6. The torsion was substantially less in the RV free wall and septal region with magnitude of 5°. During the cardiac cycle, an apicobasal displacement of 1.2 cm was also noted in the model.

#### 3.1.4. Hemodynamics

Table 1 lists the hemodynamic quantities measured at the third cycle of the biventricular model, compared against the normal range of healthy humans.

**Table 1:** Hemodynamics quantities obtained from the standard healthy model and clinical values from normal human. (Syst. - Systolic, Diast. - Diastolic).

**Quantity**	**Standard model**	**Normal human range (mean ±standard deviation)**	**References**
LV End diast.volume	148.39 ml	150 ± 31 ml, range 82–218 ml	Hudsmith et al., 2005
LV End syst. volume	71.02 ml	47 ± 15 ml, range 18–82 ml	Hudsmith et al., 2005
LV Ejection fraction	52.14%	69 ± 6 %, range 57–81%	Hudsmith et al., 2005
		57 ± 6%	Di Donato et al., 2006
LV stroke volume	77.38 ml	104 ± 21 ml, range 57–150 ml	Hudsmith et al., 2005
RV End diast. volume	144.70 ml	173 ± 39 ml, range 78–256 ml	Hudsmith et al., 2005
RV End syst. volume	67.05 ml	69 ± 22 ml, range 20–118 ml	Hudsmith et al., 2005
RV Ejection fraction	53.7%	61 ± 6 %, range 47–73%	Hudsmith et al., 2005
RV stroke volume	77.65 ml	104 ± 21 ml, range 52–128 ml	Hudsmith et al., 2005
Syst. aortic pressure	126.71 mmHg	113 ± 18 mmHg	Tandri et al., 2006
Diast. aortic pressure	75.15 mmHg	65 ± 9 mmHg	Tandri et al., 2006
Syst. pulmonary pressure	34.46 mmHg	28 ± 7 mmHg	Lankhaar et al., 2006
Diast. pulmonary pressure	13.44 mmHg	10 ± 3 mmHg	Lankhaar et al., 2006
LV End diast. pressure	10.57 mmHg	5–12 mmHg	Braunwald et al., 1961
RV End diast. pressure	4.23 mmHg	0–5 mmHg	Braunwald et al., 1961

The pressure-volume (PV) loop plots in Figure 3 depict the commonly observed rectangular shape for these loops. This indicates that the four cardiac cycle phases were simulated: isovolumic contraction, ejection, isovolumic relaxation, and filling. As the model reached stable cycles, the stroke volume of both ventricles equalized, as shown in the PV loop and Table 1. At maximum ejection rate, aortic velocity was 3.18 m s^−1^, whilst the pulmonary velocity was 2.94 m s^−1^, as shown in Figure 3. The mitral velocity was 1.04 m s^−1^ during peak filling rate, whereas the tricuspid inlet velocity was 1.07 m s^−1^.

**Figure 7:**
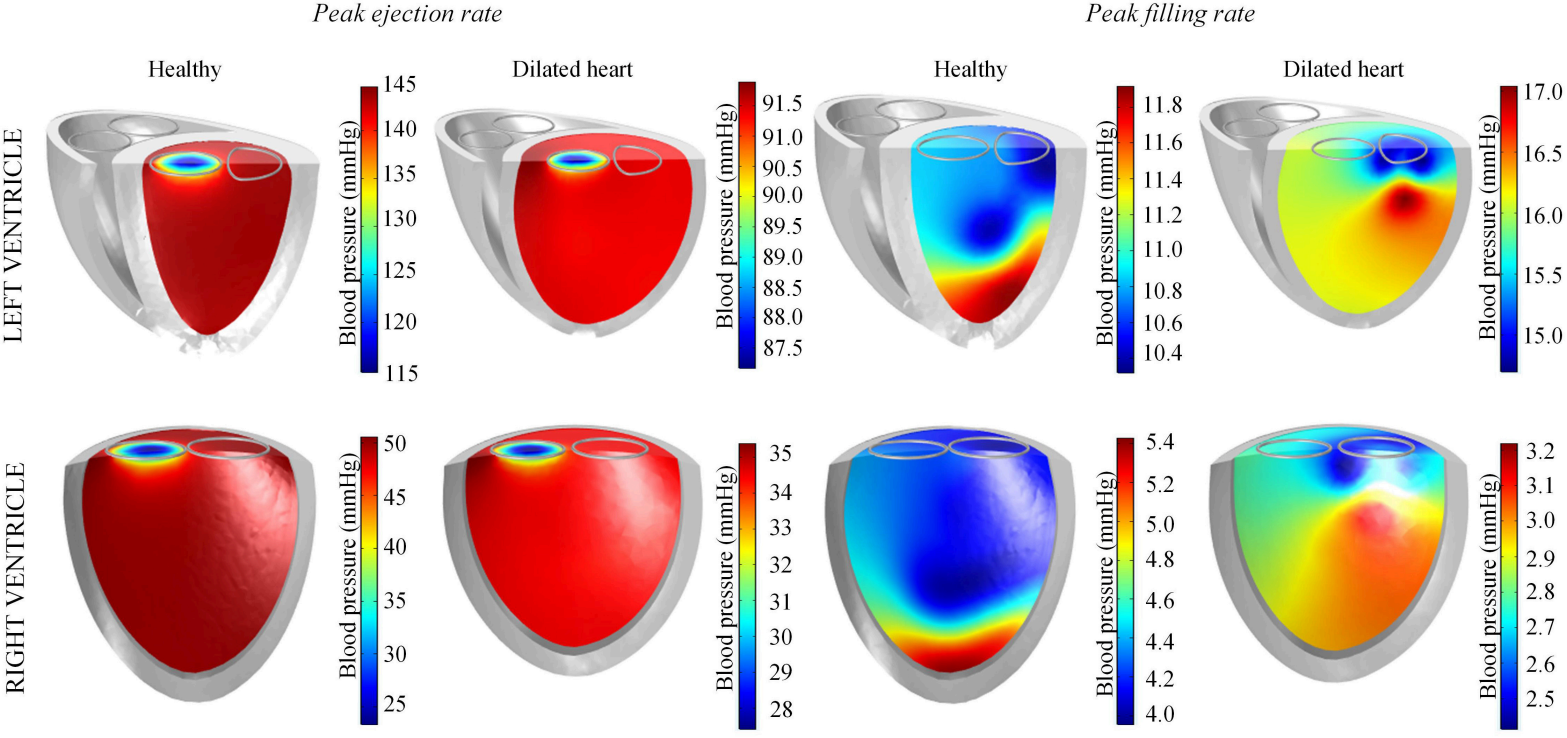
Blood pressure distribution in the left and right ventricles of the standard healthy and dilated heart models taken at timepoints of peak ejection and filling rates.

During peak ejection rate, a pressure gradient existed predominantly at the outlets (Figure 7). The pressure difference between the outlet and the inner cavity region were 31.3 mmHg and 28.9 mmHg in the LV and RV, respectively. During the filling phase, LV and RV pressure distributions showed more dispersed patterns, with the lowest pressure at the vortex centers. The LV, RV, aortic and pulmonary arterial pressure waveforms rose and dropped at nearly the same time (Figure 6). The time to peak pressure for the LV and RV also appeared to occur at a similar time, indicating a synchronous behavior. Similarly, peak filling rates occurred in near synchrony, where the time of ejection start and end occurred at the roughly the same time (outflow rates plot Figure 6).

The mean kinetic energy ($\bar{KE}$) of blood in the ventricles was computed using a similar method to Carlsson et al. (2012):

(34) $$\begin{array}{r} \bar{KE}=\rho_{f}\int |\vec{v_{f}}{|}^{2}\;dV_{\Omega_{f}} \end{array}$$

where ρ_*f*_ is the blood density, *v*_*f*_ blood velocity, and *dV*_Ω_*f*__ the ventricular fluid cavity. $\bar{KE}$ of both ventricles is shown in Figure 6C. $\bar{KE}$ was near zero prior to the start of the first contraction as the blood velocity magnitude was near zero, and was largest during the ejection phase of the cardiac cycle with the LV showing the larger magnitude of $\bar{KE}$. During isovolumic relaxation, $\bar{KE}$ dropped and rose again during the subsequent filling phase. As filling phase ceased, $\bar{KE}$ gradually declined but never dropped to near zero. Consequently, the maximum $\bar{KE}$ of the subsequent cycle's ejection phase was higher than the first cycle's.

The average vorticity, $\left|\bar{\omega }\right|$, was also measured by averaging the vorticity magnitude across the entire ventricular cavities. Vorticity, $\vec{\omega }$ was obtained via the the curl operator on the blood velocity vectors:

(35) $$\vec{\omega }=\nabla \times \vec{v_{f}}$$

The $\left|\bar{\omega }\right|$ waveform showed two main peaks in both LV and RV (Figure 6D): (1) during the ejection phase and (2) during the filling phase. The filling phase exhibited a larger magnitude of $\left|\bar{\omega }\right|$, which is also qualitatively visible in the snapshots of Figure 3 with the blood swirling motion. Following ventricular contraction, $\left|\bar{\omega }\right|$ increased momentarily before dropping significantly, but then increased again during the filling phase. Overall, the RV and LV $\left|\bar{\omega }\right|$ showed comparable magnitudes and waveform phases throughout the cardiac cycle, indicating synchronized behavior.

### 3.2. Mesh convergence

Mesh sensitivity analysis showed good convergence (< 5%) for global characteristic quantities, *C*_*q*_, of the mechanical and fluid dynamics, as summarized in Table 2. A more local measure, $\%\bar{RMS}$ comparing normal and finer meshes, revealed a good convergence (< 5%) for local electrical activation time, myocardial displacement, and fluid pressure at the time of peak LV ejection and peak LV filling rate. Whilst the fluid velocity showed a good $\%\bar{RMS}$ convergence at the time of peak LV ejection between the two meshes, the same measure was slightly higher at the time of LV filling rate.

**Table 2:** Mesh convergence analysis: comparison of characteristics quantities and $\%\bar{RMS}$ comparing normal and finer meshes.

**Metric**	**% Difference of characteristics quantities, *C*_*q*_, between normal and finer mesh**
Myocardial	0.292
displacement (cm)	
Fluid velocity (m/s)	1.636
Fluid pressure (mmHg)	0.0959
**Metric**	$\%\bar{RMS}$
Electrical activation time	3.47
Myocardial displacement at time of peak ejection rate	4.64
Myocardial displacement at time of peak filling rate	4.48
Fluid velocity at time of peak ejection rate	3.98
Fluid velocity at time of peak filling rate	7.25
Fluid pressure at time of peak ejection rate	0.012
Fluid pressure at time of peak filling rate	0.067

### 3.3. LVAD simulations

The model simulated the general impact of LVAD on the failing heart as well as, the impact of pump speed, reproducing ventricular collapse, when mitral stenosis was introduced.

#### 3.3.1. Heart failure without LVAD

By reducing LV contractility and inducing heart dilation, the LV ejection fraction (EF) dropped to 16.5% and the LV end-diastolic volume (EDV) increased to 299 ml. EF was also reduced in the healthy RV to 40%. The LV pressure, along with aortic pressure, dropped to a peak systolic pressure of merely 89 mmHg, whilst the RV peak systolic pressure reached 32 mmHg.

The qualitative behavior is shown in the snapshots of Figure 3 and the Supplementary Animation. Overall, minimal change was noted in the electrical activation sequence. The most obvious LV changes are a weaker contraction, blood vortices persisting even during systole, and a slower peak aortic ejection. On the other hand, the RV showed minimal changes relative to the standard model.

#### 3.3.2. Low LVAD pump speed

**Figure 8:**
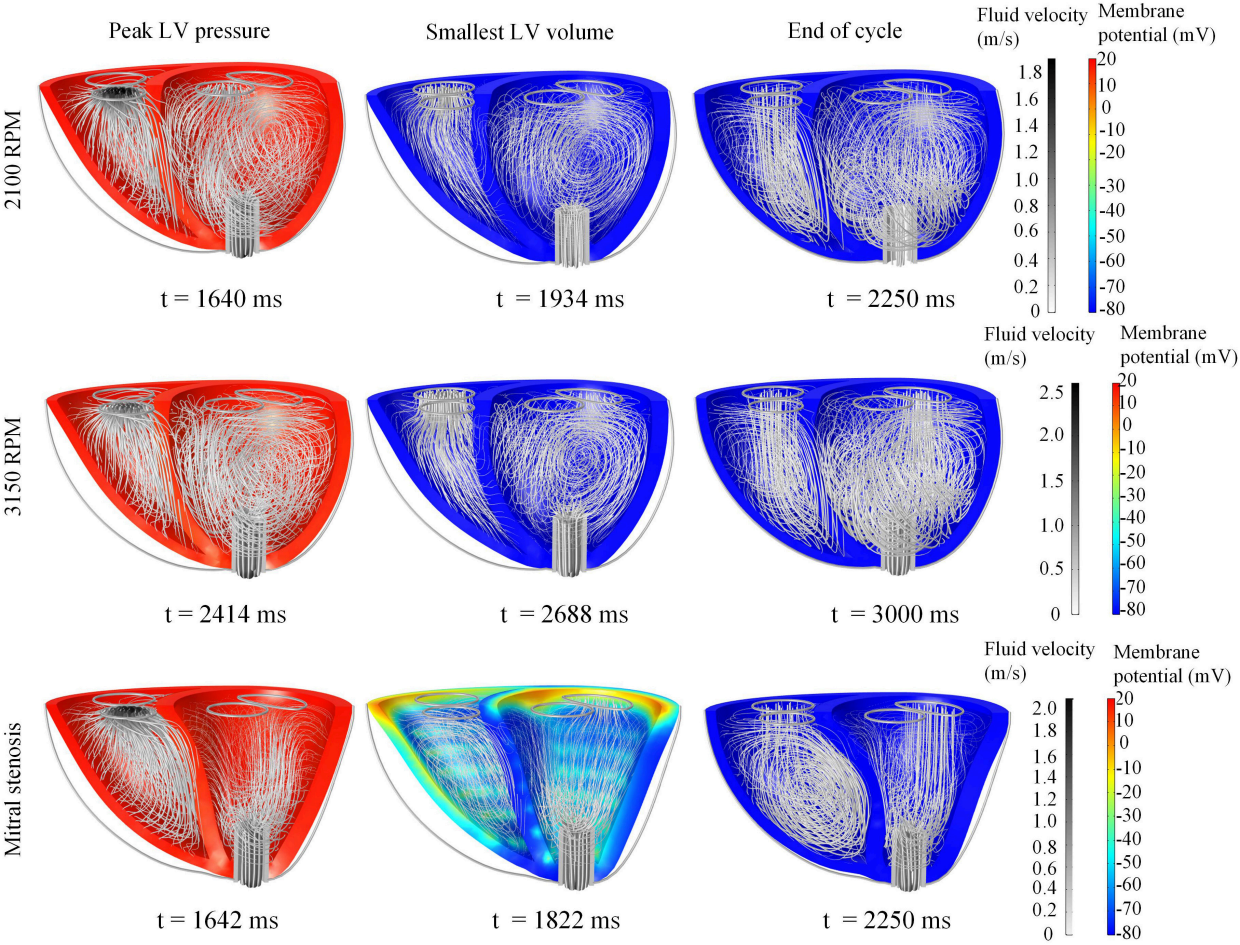
Snapshots of the biventricular model with LVAD, depicting the membrane potential, myocardial movement and fluid velocity streamlines at the times of peak LV pressure, smallest LV volume, and end-diastole. The snapshots are taken at the third cycle of stable 2,100 RPM pump speed **(Top)**, fourth cycle following speed increment to 3,150 RPM **(Middle)**, and third cycle of mitral stenosis **(Bottom)**. Streamline density is proportional to the blood velocity magnitude. The animations of these snapshots are available in Supplementary Animation 3 for the 2,100 RPM setting, Supplementary Animation 4 for speed increment setting and Supplementary Animation 5 for mitral stenosis case.

Snapshots of the fluid streamlines in Figure 8 during systole show that the aortic ejection persisted with the LVAD, albeit at lower velocity magnitude. Due to the presence of two outlets during systole, a low speed vortex region was observed in the LV free wall region. The smallest LV volume occurred at about the same time as the initiation of ventricular filling. As the LV was being filled, a fluid vortex formed at the LV free wall region and traveled toward the apex. Toward the end of the filling phase, the vortex separated into two main vortices: one near the basal LV free wall and one near the cannula. The RV showed typical systolic and diastolic streamlines as was seen in the healthy model. Throughout the cycles, the LV endocardium was well out of contact with the cannula.

**Figure 9:**
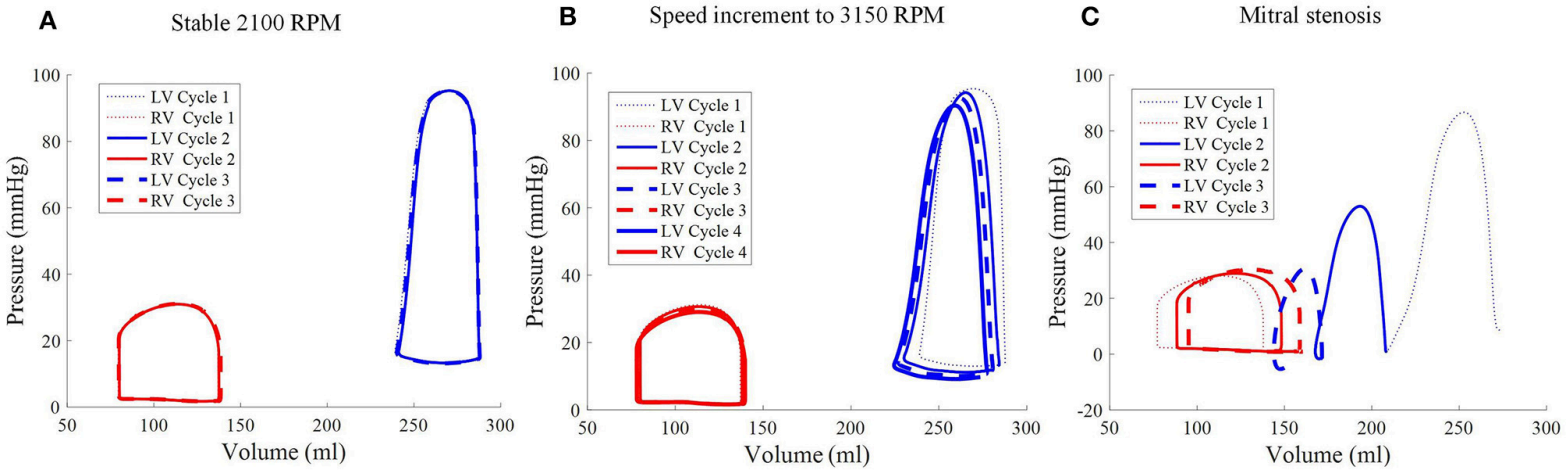
Pressure-volume loops of the biventricular model with LVAD taken at stable 2,100 RPM pump speed **(A)**, speed increment to 3,150 RPM **(B)**, and mitral stenosis **(C)**. The loop shape transformed following the speed increase (cycle 2 onwards of middle figure) and introduction of mitral stenosis **(C)** from cycle to cycle.

**Figure 10:**
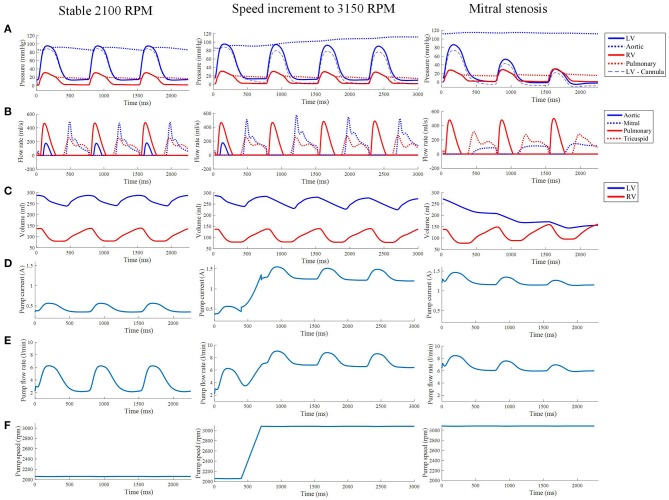
**(A)** The LV, aortic, RV and pulmonary arterial pressure waveforms from the third cycle of stable 2,100 RPM pump speed, speed increment to 3,150 RPM, and mitral stenosis. **(B,C)** The aortic, mitral, pulmonary and tricuspid flow rate waveforms **(B)** along with ventricular volume waveforms **(C)** with similar LVAD settings as the top row. The pump current **(D)**, pump flow rate **(E)**, and pump speed **(F)** waveforms from the third cycle of stable 2,100 RPM pump speed, speed increment to 3,150 RPM, and mitral stenosis.

**Figure 11:**
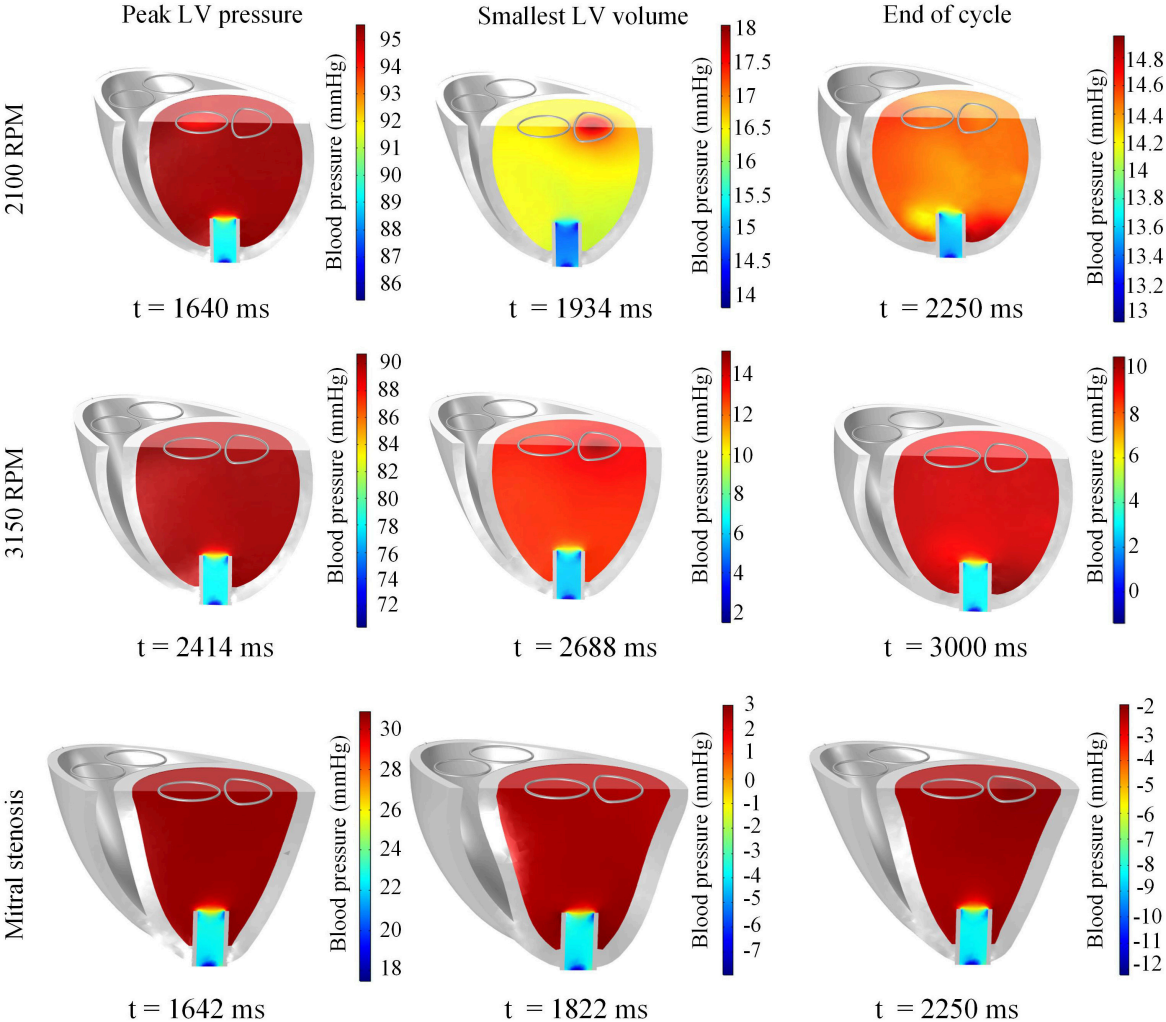
Snapshots of the biventricular model with LVAD depicting the LV blood pressure distribution at the times of peak LV pressure, smallest LV volume, and end-diastole. The snapshots are taken at the third cycle of stable 2,100 RPM pump speed **(Top)**, fourth cycle following speed increment to 3,150 RPM **(Middle)**, and third cycle of mitral stenosis **(Bottom)**.

Throughout the three cycles, the PV loops overlapped, indicating stability (Figure 9A). It can be seen that aortic ejection was preserved at this pump speed, although lower than the pre-LVAD setting. The aortic pressure peaked at 95 mmHg and reached a minimum of 86 mmHg, displaying less pulsatility (Figure 10). The LV pressure waveform observed at the LV base and the pressure waveform at the cannula showed synchronous characteristics, and were nearly identical. This was also illustrated in the LV pressure distribution of Figure 11 where the LV pressure is lowest at the cannula. The LV pressure gradients were highest during the systolic phase (≈ 9 mmHg) and lowest at diastole (≈ 2 mmHg).

The LVAD model also provides measures of pump electrical current, pump flow rate and speed. With the proportional controller applied, the pump speed was nearly constant with very insignificant (< 1%) drops during systolic contraction. The pump motor current showed a bump to 0.57 A from a baseline of 0.35 A, coinciding with increased pump flow rate to a peak of 6.2 L min^−1^ from a baseline of 2.2 L min^−1^. Qualitatively, it can be seen that the pump motor current and flow rate waveforms coincided with the LV pressure waveform due to direct correlation with Δ*P* in Equations (25)–(28).

#### 3.3.3. Sudden increment of pump speed

A sudden increase of pump speed to 3,150 RPM immediately resulted in an increased aortic pressure, causing a cessation of aortic ejection in the subsequent cycles (Figure 10). Both peak LV and RV pressures also gradually dropped from cycle to cycle as the high pump speed was maintained. Moreover, LV pressure gradient between the cannula and ventricular base increased, as illustrated in the pressure snapshots of Figure 11. At the end of the third cycle, the pressure became negative in the cannula region (Figure 11).

As shown in Figure 10, the increase in speed was accompanied by an immediate increase in the pump flow rate from the previous baseline of 2.2 L min^−1^ to a new baseline of 7 L min^−1^. During systole, the pump flow rate peaked at ≈9 L min^−1^ as opposed to 6.2 L min^−1^ at the lower, 2,100 RPM, speed. Consequently, the pump current also elevated to a baseline of 1.2 A with a maximum current of 1.5 A.

With the cessation of aortic ejection, the blood flow streamlines during systole were directed toward the cannula at a higher velocity, as shown in the snapshots in Figure 8. Slight swirling was also observed in the region surrounding the cannula. Similar to the low speed setting, the smallest LV volume occurred at about the same time as the mitral filling phase. At end-diastole, both ventricles exhibited fluid vortices. The cannula and the LV endocardium were not in contact throughout the simulation (Figure 8).

**Figure 12:**
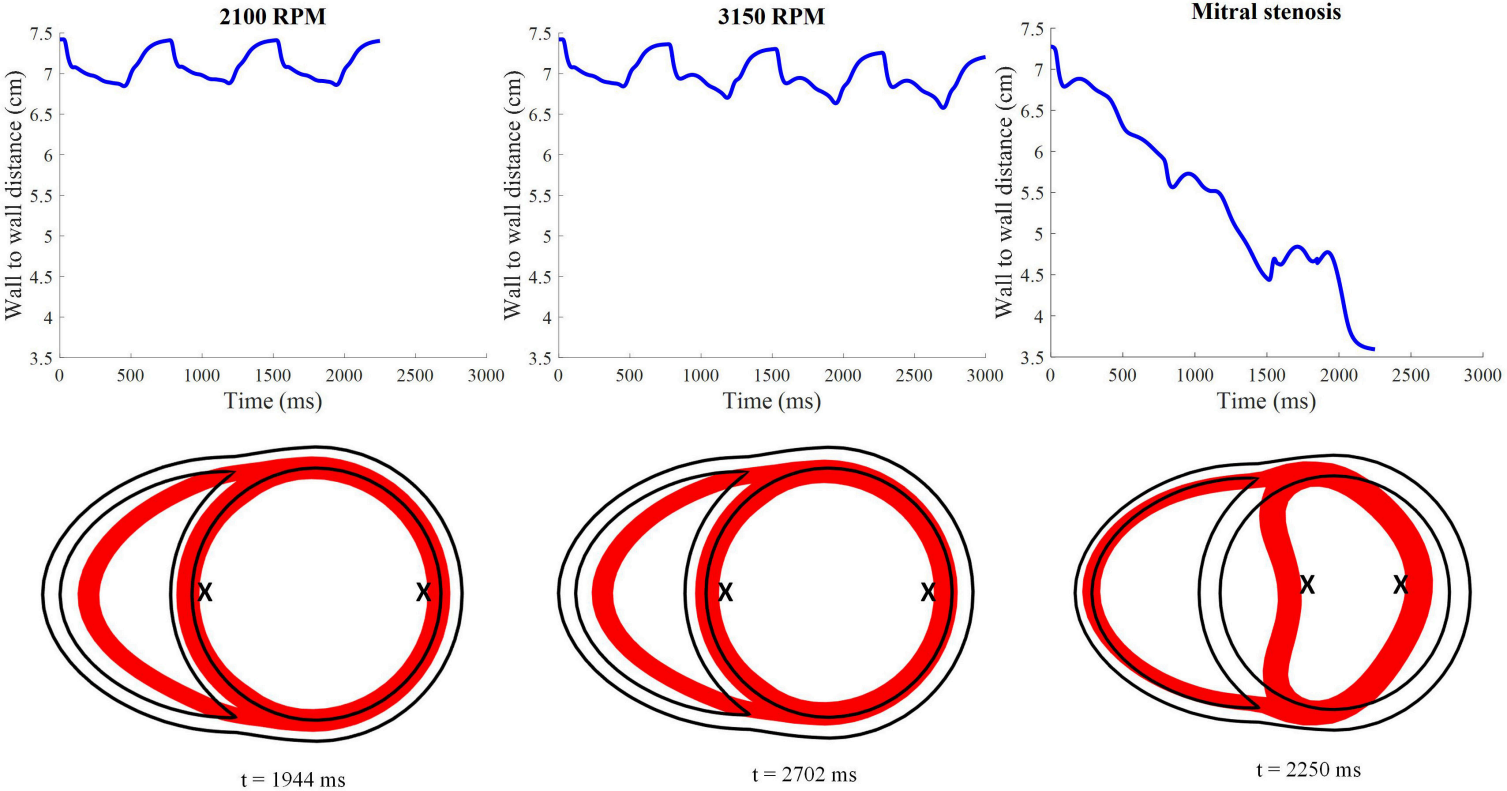
Distance between the LV free wall endocardium and LV septal endocardium midway between the apex and the base. The lower 2D plots depict the cross-section of the ventricles taken at midlevel along the apicobasal direction for all LVAD simulation settings. The red surface indicates the myocardial cross-section at time of smallest LV whilst the black outline indicates the myocardial cross-section at time *t* = 0. “X”; denotes the two points used to calculate the wall to wall distance.

As the cycles went on, the LV pressure-volume loops gradually became more triangular (Figure 9B). The LV EDV also gradually reduced, along with overall LV blood pressure characteristics. The increased speed also appeared to improve RV function, with enhanced ejection fraction and lower peak RV pressure. The de-congested LV also resulted in a small, yet gradual, wall collapse as quantified by the decrease in distance between the LV free wall and septum (Figure 12).

**Figure 13:**
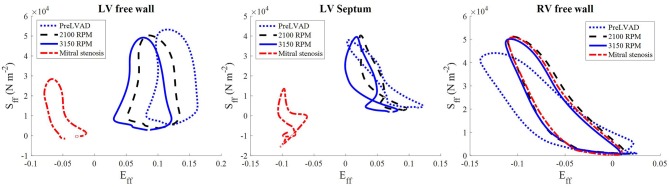
The stress-strain loops of the LVAD simulations at the third cycle (*t* = 1,500–2,250 ms) of stable 2,100 RPM pump speed **(Left)**, speed increment to 3,150 RPM **(Middle)** at (*t* = 2,250–3,000 ms), and mitral stenosis (*t* = 1,500–2,250 ms) **(Right)**. S_ff_ is the 2nd Piola-Kirchoff stress and E_ff_ is the Green-Lagrange strain tensor components along the fiber direction.

The stress-strain loops measured at the epicardial sites along the mid-apicobasal distance, LV and RV free walls and septum, suggest a potential change in regional work in the ventricles. The fiber components of the stress-strain loop showed a gradual shift to the left as the speed increased, especially for the LV free wall (Figure 13). On the contrary, minimal change was noted in the RV stress-strain loops.

#### 3.3.4. Mitral stenosis with LVAD

In the first cycle with mitral stenosis, the systolic measures did not appear to be affected by the stenosis. During diastole, the introduction of mitral stenosis severely impeded the inflow into the LV, resulting in an immediate imbalance between the pump flow rate and input flow rate into the pump. As such, the LV collapsed as the cycles continued.

The major impact of mitral stenosis appeared from the second cycle onwards, in which the ejected blood was not replenished in the LV for the subsequent cycles, resulting in a lower LV EDV in subsequent cycles. Consequently, the LV pressure generated from the second cycle onwards was significantly lower (52.9 mmHg) than prior to mitral stenosis (86.6 mmHg). The LV pressure dropped even further in the third cycle to 30 mmHg, nearly similar to the RV pressure, as shown in Figure 9C.

The collapsing LV also resulted in the LV PV loop to be smaller than the RV (Figure 9). The LV PV loop also shifted downwards and leftward toward the negative pressure region. The RV PV loop was also affected, as it shifted to the right, since the RV started to dilate with a slight increase in peak RV pressure. In addition, the fiber direction stress-strain loop, exhibited in Figure 13, also shrank significantly for the LV free wall and septum across all strain and stress tensor components.

The snapshots in Figure 8 showed an obvious shift in the septum orientation toward the LV. Mitral flow was noted to initiate earlier, and last for a longer duration (Figure 10). The smallest LV volume occurred at an earlier phase of the cardiac cycle, compared to previous settings. During end-diastole, the fluid vortices were notably absent in the LV, and only present in the RV. The snapshots of LV blood pressure distribution in Figure 11 also revealed greater negative pressure, with the filling phase showing an entirely negative pressure throughout the whole chamber. With the ventricular wall collapsing into the LV, it can be seen that the endocardium was in contact with the cannula throughout the cardiac cycles. The distance between LV free wall and septum was reduced significantly in the presence of mitral stenosis (Figure 12).

The reduction in LV pressure also manifested in a reduced peak pump flow rate and pump current (Figure 10). Nevertheless, the baseline pump flow rate and current were maintained at approximately 6 L min^−1^ and 1.3 A, respectively. The waveforms also became less pulsatile with the onset of ventricular collapse.

## 4. Discussion

In this study, we developed a cardiac multiphysics modeling framework for biventricular electrical, mechanical and fluid dynamics physics in a healthy heart. The modeling framework was then applied to simulate a failing heart with LVAD support. This framework can be applied in future pre-clinical investigations of device performance under various pathological conditions.

### 4.1. Standard biventricular model

#### 4.1.1. Electrical characteristics

The simulated LV activation pattern is nearly radial and the RV activation pattern is nearly tangential ending at the basal region, whilst the interventricular septum is predominantly activated in a left to right sequence. The first epicardial breakthrough occurred in the RV due to its thinner wall structure. As such, the model's electrical activation reproduced normal human electrical activation sequence (Durrer et al., 1970). Furthermore, the total duration of electrical activation, 94 ms, falls within the range of the QRS duration in healthy humans (80-120 ms), which corresponds to the ventricular activation time (Guyton and Hall, 2006). The model also reproduced the transmural APD gradient observed in the healthy myocardium. In addition, AP repolarization began from the epicardium and ended at the endocardium, the opposite direction to the activation. Such behavior has been recorded in ventricular tissue strips from rejected donor hearts by Glukhov et al. (2010).

The Purkinje fiber network implementation enabled simulation of a healthy activation sequence, and can be applied in future studies to simulate diseased activation such as bundle branch block by simply disabling select branches of the Purkinje tree. The Purkinje fiber APD is longer than that of the nearby contractile myocardium to prevent retrograde activation (Vigmond and Stuyvers, 2015). Although current flow from the myocardium to the Purkinje fibers was not included in this study, retrograde activation can be considered in the future. For example to simulate ectopic beat re-entry, an additional current could be added to the Purkinje AP model of Equation (2) (Vigmond and Stuyvers, 2015).

AP propagation physics was formulated in the material frame to emulate gap junction controlled propagation as described in our previous work (Bakir and Dokos, 2015). Since the resistivity of the gap junctions is higher than the intracellular space of cardiomyocytes, the myocardial substrate can be treated as network of interconnected resistors whose total electrical resistance remains unchanged as the structure deforms. This phenomenon has been experimentally observed in a number of animal species by Penefsky and Hoffman (1963), who measured papillary muscle conduction velocity while they were stretched. For this reason, formulating the electrical propagation in the material frame represents a more suitable approach. Although, the use of material frame-based formulation may have an insignificant effect on rhythmic activation patterns, we have shown that in more complex electrical activation scenarios, such as arrhythmias, the effect is prominent (Bakir and Dokos, 2015).

#### 4.1.2. Mechanical variables

The predicted apex to base shortening of 1.2 cm is within the range measured in healthy humans (Alam et al., 1990). The torsion was predominantly within the LV due to its larger muscle mass, as opposed to the RV. The LV wall torsion magnitude was within the range reported by Henson et al. (2000). On the other hand, RV torsion measures have been under-reported in the literature. Nonetheless, it is expected that the RV exhibits lesser twist as its thinner wall accommodates lesser circumferential fibers and more oblique fibers (Pettersen et al., 2007). The RV also restricted the septal motion generating lesser torsion in the septum as opposed to the LV free wall. Thus, the common healthy ventricular mechanics were observed in our biventricular model.

Stress-strain loops are indicators of regional ventricular work, which may be correlated to myocardial oxygen consumption and potential structural remodeling (Russell et al., 2012). Our model predicted a relatively large and box-shaped loop for the LV free wall, indicating a greater work load on this section of the myocardium to eject blood, compared to those at the LV septum. This is in agreement with a clinical study that reported regional work is larger in the LV free wall compared to LV septum (Russell et al., 2012). The predicted RV free wall stress-strain loop was smaller than the LV free wall. This is due to the lower afterload pressure imposed on the RV, even though both ventricles produced a similar cardiac output.

#### 4.1.3. Hemodynamics

Global hemodynamics measures predicted by our model were similar to normal quantities reported in healthy humans. The simulated LV ejection fraction and stroke volume were on the lower end of normal ranges, possibly due to the smaller amount of myocardial thickening exhibited by our model. We speculate that this in turn was due to our assumption of transverse isotropy. A multiscale model by Washio et al. (2013) includes tissue level structure in its myocardial mechanics formulations, enabling modeling of cleavage planes. This facilitates sheet sliding, which helps to eject an additional 7.5 ml of blood compared to modeling without this level of detail. However, inclusion of realistic microstructure will hugely increase computational workload. Our fixed constraint at the base and lack of geometrical outflow tract may also impede further motion of the ventricles, thus rendering a lesser amount of myocardium usable for volume displacement, and therefore blood ejection.

Our simulations predicted a slightly higher pressure gradient within the LV (≈ 31 mmHg) than typical values reported for healthy humans (< 30 mmHg), even though the outlet boundary area is within the healthy human range (Westaby et al., 1984; Geske et al., 2011). Nevertheless, our predicted value does not exceed the severe obstruction threshold of 50 mmHg defined by Geske et al. (2011). As a consequence, the predicted peak velocity of inlet and outlet flows are also higher than normal (Mowat et al., 1983). We hypothesize that this disparity is due to our simplified generic structure with a flat ventricular base, whilst in reality the structure is slightly angled and funnel-like, which may help direct the flow (Greenbaum et al., 1981). This issue could be resolved when the framework introduced here is applied to an anatomically realistic structure for future patient-specific simulations.

The filling phase FSI has been of considerable interest in cardiac mechanics research, particularly the formation of a vortex ring and its possible link to cardiac efficiency (Hong et al., 2008). In our healthy simulation, a vortex ring formed in each of the ventricles during the early filling phase, growing in size as they approached the apex. As the filling ceased, the vortex ring moved to the basal region near the outlets. This simulated LV behavior matched the description reported in the echocardiographic study of Hong et al. (2008), which attributed the vortex to the occurrence of a shear layer between the high speed mitral jet and the low speed blood movement in the cavity, causing the mitral jet to roll up, forming a vortex. These investigators reasoned that the vortex helps to maintain kinetic energy of the blood prior to ejection.

Blood $\bar{KE}$ and $\left|\bar{\omega }\right|$ have been proposed as potential markers for predicting cardiac dysfunction (Carlsson et al., 2012). The systolic and diastolic peaks of $\bar{KE}$ have been calculated clinically from 4D flow MRI data (Carlsson et al., 2012). Whilst our model's RV $\bar{KE}$ is similar in behavior to that reported by Carlsson et al. (2012), this is not the case for the LV. The clinical measurement revealed the LV $\bar{KE}$ is larger during diastole than during systole, which is not seen in our simulations. Carlsson et al. (2012) argues that two mechanisms contribute to the diastolic $\bar{KE}$: elastic recoil of the myocardium and the displacement of the ventricular base into the position previously occupied by the atria. Since the atrial structure is not incorporated in our model, this latter effect cannot be simulated.

The $\bar{KE}$ never dropped to zero, which can be attributed to the vortex present in the chambers during diastole. The vortex has been suggested to aid energy and momentum transfer in the fluid, and the vorticity magnitude has been proposed as a measure to predict LV diastolic dysfunction caused by ventricular interdependency in cases of RV failure (Schäfer et al., 2016). Both RV and LV $\left|\bar{\omega }\right|$ magnitude was largest during the filling phase as expected, due to the presence of rotating flow observed in Figure 6. A slight bump was observed during systole, which can be caused by the fluid being pushed against the flat fixed basal boundaries. It should be noted that the atrial contraction will generate an additional vortex, affecting $\left|\bar{\omega }\right|$ (Schäfer et al., 2016).

In comparison to the passive filling vorticity measured by Schäfer et al. (2016) in healthy human LVs (≈ 30 1/s), LV $\left|\bar{\omega }\right|$ simulated by the standard model was higher. We hypothesize that this discrepancy is due to filtering out the larger vorticity magnitude (> 70 1/s) during clinical image analysis, in addition to the lack of internal ventricular structures such as valves and papillary muscles in our model geometry, which could affect the vortex strength. Although both $\bar{KE}$ and $\left|\bar{\omega }\right|$ did not entirely replicate realistic measurements, the details presented here could provide basic insights on how disease states affect blood energetics. More accurate measures would be obtained should a more realistic structure be implemented.

### 4.2. LVAD simulation

We simulated the success of the LVAD in restoring the aortic pressure in a failing heart. An immediate reduction in the native aortic flow rate was also observed. Moreover, our computational simulations demonstrated that an imbalance of pump flow rate and LV filling rate can result in ventricular collapse, a phenomenon known as suction, which can impede the pump's performance. As such, our modeling framework can be applied as a simulation tool to optimize LVAD design, as well as predict and investigate any clinical risks for various heart conditions prior to clinical studies, considering LVAD is designated as high risk (Class III) device by the US FDA.

By simply adjusting the geometry and myocardial contractile strength parameter, *k*_*Ta*_, the standard model was modified to simulate typical characteristics of LVAD recipients. The diseased heart model's LV EF (16.5%), LV internal diameter (7.4 cm) and LV EDV (299 ml) are within the ranges observed in severe dilated heart failure patients (Dandel et al., 2008). Therefore, the standard model can be easily modified to simulate behaviors of relevant diseases.

#### 4.2.1. Impact of LVAD on hemodynamics

Computational electrical-FSI simulations offer advantages over most imaging modalities to investigate fluid velocity profiles in the presence of LVADs. This is due to their superior resolution and no risk of image artifacts brought by the metallic implants (Carr et al., 2010). Analysis of the fluid streamlines can be beneficial to determine risk of thrombosis associated with cannula design and placement, as was performed in the 2D modeling study of Ong et al. (2013).

Following LVAD placement, our model displayed an immediate increase in aortic pressure due to the additional input on the systemic circulation by the LVAD outflow. As such, higher LV pressure was needed to enable opening of the aortic outlet. However, with a weaker contractility, the LV was not capable of generating sufficient pressure to overcome the restored aortic pressure. This explains the reduction in the aortic flow rate and aortic ejection duration. With further increase in pump speed, the aortic pressure rose more, resulting in a complete cessation of aortic ejection. Aortic valve closure is a known complication in many LVAD recipients as it may result in valve fusion, thus requiring speed adjustment as a prevention (Rose et al., 2000). Therefore, using computational simulations under patient-specific settings, it is possible to determine whether a safe window of pump speed operation exists for a certain ventricular state prior to clinical implantation.

Our simulations predicted separation of pressure waveforms measured at the LV base and cannula as the pump was maintained at a higher pump speed. Such behavior has been observed in a canine experiment where separation of pressure waveforms was associated with suction or overpumping characteristics (Salamonsen et al., 2015). Following implantation, the normally rectangular LV pressure-volume loop transformed into a more triangular shape. This was due to the loss of isovolumic phases, since the LVAD model is based on a continuous flow pump. The triangular shape became more prominent at a higher pump speed as the LV pressure generation capability was negated by the stronger LVAD flow rate.

Under reduced LV filling induced by mitral stenosis, the LV pressure-volume loop was severely depressed because the pump flow rate was larger than the inflow rate supplied by the left atrium. Therefore, the LVAD emptied the LV, causing it to collapse. Consequently, native heart pumping capability was impaired, as predicted by the end-systolic pressure volume relation (ESPVR), which linearly relates the maximum pressure that can be generated with the LV volume. During LV collapse following mitral occlusion, the LV is held at an extremely low volume, so, according to the ESPVR it is expected that the generated LV pressure is low (Guyton and Hall, 2006).

Increasing the LVAD pump speed appeared to result in diminished diastolic vortex formation, as shown by the fluid streamlines in Figure 8. This is further aggravated in the collapsed LV induced by mitral stenosis. We speculate that this change can be attributed to the LV geometry becoming less ellipsoidal, which could reduce space for the vortex to form. This effect is especially exaggerated in the collapsed LV.

#### 4.2.2. Impact of LVAD on myocardial mechanics

Following the increase in LVAD pump speed, our simulations revealed that the stress level experienced by the LV wall gradually dropped from cycle to cycle. The stress-strain loops assessed along the fiber direction exhibited a leftward trend, and the local strain was reduced, which are the result of LV decongestion by the LVAD.

On the contrary, our model predicted only a small drop in RV stress level following LVAD intervention, whilst the RV strain components were unchanged. This is similar to predictions by the modeling study of Sack et al. (2016) that RV stresses remain unaltered following LVAD intervention. The improvement in the RV PV loop under LVAD support, shown by our simulation, may only be due to the alteration in septal stresses and strains due to the unloaded LV. As such, only a slight PV improvement was noted and the RV free wall mechanical work remained unchanged.

When LVAD support was simulated in the presence of mitral stenosis, the LV was substantially drained and all LV stress-strain tensor components reduced significantly, indicating a severe reduction of LV mechanical regional work. The LV chamber lost its ellipsoidal geometry and its diameter was reduced significantly. At the apex, mechanical contact was observed between the endocardium and the cannula. If such a suction event is not alleviated, myocardial injury may occur and could trigger arrhythmia (Vollkron et al., 2007).

#### 4.2.3. Behavior of LVAD variables

In our simulations, pump motor current and flow rate waveforms approximately followed the LV pressure waveform. During contraction, pump flow rate increases due to reduced pressure difference between the aorta and the LV that needs to be overcome by the pump. The increase in pump flow results from the increased hydraulic loading on the pump impeller, as noted by the torque transfer formulation in Equation (27) (Lim et al., 2010). Therefore, the pump motor current has to increase as well to generate more torque to overcome the increased hydraulic loading.

At higher pump speed, the simulation predicted loss of pulsatility (Figure 10), which coincided with the loss of peak ventricular pressure. Since the pump produces stronger torque at higher speed, the increased hydraulic loading can be easily overcome by the pump without requiring an extensive increase in pump current during systole. The collapsed LV imposed greater resistance to the pump flow rate, causing the pump flow pulsatility to be severely weakened.

Pump current waveforms have been used as a measure to detect suction events in the heart under LVAD support (Yuhki et al., 1999). As the pump current waveform shows good agreement with the LV pressure waveform, this measure can be used to detect suction events, heart rate and aortic valve closure. The pump current is a non-invasive measure that is readily available in the pump. Furthermore, it does not suffer baseline drifting, typically experienced by pressure sensors (Troughton et al., 2011). Our biventricular model can be applied as a simulation tool to aid design and test better controllers capable of detecting these events and providing counter measures, such as lowering the pump speed or alerting medical personnel (Stevens et al., 2014; Robertson et al., 2017), before the patient situation gets worse.

Overall, we observed a stable pump speed throughout the simulation, with the pump speed was nearly similar to the target speed, ω_*set*_ as shown in Figure 10F. Whilst Lim et al. (2010) employed a proportional-integral (PI) controller to maintain the speed, we did not model the integral component. Nevertheless, we did not observe any major discrepancy in the pump speed relative to ω_*set*_, indicating a sufficient controller function for the simulation.

#### 4.2.4. Impact of LVAD on the RV

Our simulations predicted a small improvement in the RV function with LVAD support, in particular a stroke volume increase and a drop in peak RV pressure to a healthy level. Further improvements were observed with an increase in pump speed. These improvements may be attributed to increased RV filling subsequent to LVAD-enhanced LV output, as well as the direct result of reduced afterload (Morgan et al., 2013). As the LV decompressed, LV filling improved and subsequently reduced the pulmonary arterial pressure. With reduced afterload, RV ejection fraction is expected to improve according to the end-systolic pressure volume relation (Maughan et al., 1984).

On the contrary, when mitral stenosis was induced in the simulation, RV dilation and increased peak RV pressure reappeared. The mitral stenosis essentially increased RV afterload, preventing more blood from leaving the pulmonary circulation. As such, RV pressure increased to counter the increased afterload. With the baseline pump flow rate barely changed and the increased afterload impeding RV outflow, the RV began to dilate. This helped push the septum further into the LV chamber as the LV collapsed.

Clinically there is considerable interest in the impact of LVADs on the RV due to the significant pool of LVAD recipients developing RV failure (Neyer et al., 2016). Nonetheless, a clinical observation by Morgan et al. (2013) noted that LVAD actually improved RV function in some patients. RV failure may be caused by an undetected RV disease that is masked by more significant LV failure. LVAD support improves the venous return to the RV; however this may aggravate the undetected RV disease as a weaker RV will be unable to cope with the improved preload. This could be investigated in future with this model to optimize LVAD settings prior to implantation.

To our knowledge, previous finite element models of heart-LVAD interaction did not consider the biventricular interaction. For example, the study by McCormick et al. (2013) expands the LV FSI model of Nordsletten et al. (2011) by adding an immersed cannula structure, but does not implement any electrophysiology physics. Other studies by Sack et al. (2016) and Heikhmakhtiar et al. (2017) simply modeled the LVAD contribution by adding a constant flow from the heart and into the aortic circuit of the four-chamber Living Heart Model (Baillargeon et al., 2014) and the Gurev et al. (2011) biventricular model, respectively. Nevertheless, the cannula itself and the fluid dynamics were not modeled in these studies.

The framework presented here offers a number of advantages over previous heart-LVAD models. In particular the capability to simulate

1. the impact of abnormal electrical activation on LVAD pump performance. As noted by Robertson et al. (2017), LVAD recipients have high arrhythmic tendency that may result in RV dysfunction and suction events.
2. pump current behavior throughout the cardiac cycle. Pump current provides a minimally invasive measure that can be utilized in future pump controller designs to predict the present state of the heart.
3. LVAD impact on the RV. This allows study of various metrics to predict vulnerability of LVAD-induced RV failure in future. Criteria could be developed based on these metrics to support the clinical decision whether an LVAD or biventricular assist device (BiVAD) should be implanted in a patient to ensure optimal management.
4. the impact of cannula shape and positioning on LVAD performance. Non-optimal cannula placement can reduce pump performance and has been suggested to induce thrombosis (Ong et al., 2013; Neyer et al., 2016).

Considering the LVAD is classified as high risk class III medical device by the FDA, we believe computational modeling via the framework introduced here can help improve device safety and efficacy prior to pre-clinical studies and clinical testing in patients with severe heart disease. Furthermore, some devices, such as the pediatric Berlin Heart LVAD, have also been approved for rare medical cases through the Humanitarian Device Exemption (HDE) rule, which relaxes the clinical efficacy requirement due to the lack of test subjects (Almond et al., 2011). Computational simulations can help fill this gap and provide a higher degree of confidence among clinicians and regulatory bodies to approve the usage of this high risk device in this cohort of patients.

With all major cardiac physics present in this model, simulations can be easily expanded for other treatments and diseases. The inclusion of basic fluid hemodynamics allows the model to simulate internal flow characteristics of the heart with various implants such as artificial valves, which cannot be presented in electromechanical simulations such as Kerckhoffs et al. (2009). On the other hand, FSI simulations with spatially-uniform contraction stress, such as Krittian et al. (2010), cannot be extended to include electrical abnormalities. Inclusion of a closed-loop circulation and biventricular structure also provide the capability to study biventricular interaction, which cannot be simulated in the previous LV fluid-electromechanics studies (Watanabe et al., 2004). As such, the model presented here provides a framework for more intensive and extensive cardiac simulations.

### 4.3. Limitations of current modeling framework

A morphologically-realistic geometry was not implemented as the main aim of this study was to demonstrate the model's capability to simulate cardiac multiphysics phenomena. An idealized geometry eases the computational load, making it more suitable for framework development. We consider this study a stepping stone for future simulations based on patient-specific anatomies, extracted from imaging modalities such as computed tomography (CT).

In terms of electrophysiology, the model presently does not include several mechanofeedback components such as the stretch-activated currents and stretch effect on the cellular membrane's caveolae. Stretch-activated channels have been proposed to be capable of triggering electrical activation via mechanical stretch alone (Sachs, 2010). However, more characterization needs to be performed to better understand the channel kinetics. The effect of stretch-activated channels can be easily added to the source term in Equation (1) in the future. Stretch may also reduce the number of caveolae structures, leading to a reduction in membrane area and hence reduced membrane capacitance. This effect has been found to slow electric conduction in the cardiac myocyte (Pfeiffer et al., 2014). Thus, this suggests that the membrane capacitance must be made stretch-dependent if such characteristics are to be modeled. Nevertheless, it should be noted that the addition of these stretch-dependencies may increase the non-linearity of the electrical physics. It is likely that these additions may not alter the predictions of most organ-level simulations as the electrical to mechanical interaction is the more crucial coupling.

The use of phenomenological AP and active stress formulations provided considerable simplification, since it reduced the number of variables required to be solved relative to biophysically-based models. This also enabled the use of larger time step steps, for more efficient computation. Mechanical dependencies of the contraction strength may be necessary for specific future studies to simulate the Frank-Starling mechanism, which was not considered in the present model. The mechanism enables the heart to pump at a greater ejection fraction should it be filled with a larger amount of blood (Guyton and Hall, 2006). Nevertheless in failing heart, the Frank-Starling mechanism is known to be absent (Schwinger et al., 1994). With mechanical dependencies, numerical instability may arise especially if a segregated-type solver is implemented (Niederer and Smith, 2008). While a fully coupled-type solvers as adapted here and elsewhere (Göktepe and Kuhl, 2010)are more robust, they tend to require larger computer processing power and memory. As such, additional tuning of the solver settings may be necessary.

Passive myocardial mechanical properties were simply assumed to be a transverse isotropic hyperelastic, whilst in reality, the myocardium exhibits orthotrophic properties (Dokos et al., 2002). Nonetheless, the transverse isotropic hyperelastic material should be able to replicate the uniaxial and biaxial mechanical testing by Demer and Yin (1983). However, if realistic shear characteristics are desired, the orthotrophic terms of Holzapfel and Ogden (2009) can be simply added to the existing strain-energy function.

Viscoelasticity is a common property of myocardial tissue, contributed by the collagenous structure and intercellular fluids, helping to damp out vibration, due to the blood flow (Cansiz et al., 2017; Quarteroni et al., 2017). In the model presented here, a Rayleigh damping component was added to damp out oscillations, where the Rayleigh damping parameters were set proportional to the mass and stiffness terms in the equation of motion. In future, incorporation of the viscoelastic components would be a more accurate way to damp out such oscillation without the need for Rayleigh damping.

Active atrial contribution to the filling phase was not considered in the present study. The atrial contraction is expected to produce an additional peak in the filling flow rate waveform, which forms the “A” wave commonly observed in Doppler imaging of the flow waveforms (Sohn et al., 1997). This atrial contraction will form an additional vortex following the early passive filling (E-wave), and will thus affect the blood kinetic energy and average vorticity plots Hong et al. (2008). The “A” waveform could be simulated by adding an active atrial function in the Windkessel atrial compliances to simulate their physiological pressure-volume relations. In addition, filling kinetic energy is dependent on the basal displacement into the spatial location, previously occupied by the atria (Carlsson et al., 2012). However, incorporating this effect into the simulation would require structural modeling of the atria as well.

Valve structure will influence the intra-ventricular blood flow profile. Furthermore, the angled position of the valves may also ease flow resistance during filling and ejection. Among LVAD recipients, tricuspid regurgitation has also been noted, in particular in those who developed RV failure, since the RV may not be able to cope with the restored systemic venous return (Hayek et al., 2014). Furthermore, aortic valve fusion is a known issue, experienced among LVAD recipients (Rose et al., 2000). Consequently, incorporating the valve structure mechanics into the multiphysics models may be necessary in future due to the high prevalence of valve failure in many ventricular diseases. Inclusion of valvular structure will likely require contact modeling, as was performed between the endocardium and cannula, as well as employing a re-meshing algorithm to overcome severe mesh distortion. Although we used laminar Navier-Stokes formulations, the turbulent form may need to be considered in future, especially in the presence of structural valves. Turbulence models have rarely been considered in multiphysics simulations, since they are more computationally expensive, mainly applied in pure fluid simulation studies (Chnafa et al., 2015).

The model mesh we have used was selected as a compromise between global convergence and computational cost. Fluid dynamics, especially during the diastolic phase, can involve more complex flow patterns, due to vortex formation and the presence of low velocity regions; these may require a finer mesh to be accurately modeled. With the present mesh setting, a converged solution was obtained for blood flow systolic characteristics, but the convergence slightly worsens during the isovolumic phases and diastolic filling. Nevertheless, qualitative and global measures such as vortex shape and flow waveforms can be sufficiently obtained with present mesh settings. In addition, changes to action potential parameters such as σ and *k*_2_, resulting in increased conduction velocity, will require finer meshing to achieve a converged conduction velocity and to prevent numerical convergence failure at large element size. Therefore, future mesh settings need to be tailored to the requirements of the study.

## 5. Conclusion

The cardiac multiphysics framework developed was applied in a biventricular structure with an idealized Purkinje fiber network utilizing a modified form of the standard cable equation, taking into account the change in Purkinje fiber radius. The Windkessel circulation was expanded by including a closed-loop circulation which linked both ventricular chambers. The model managed to simulate healthy LV and RV electrical activation sequences, mechanical behavior as well as global hemodynamics. This highlights the model's capability for future biventricular modeling work. The model was also tested to simulate LVAD support and impact of the pump on ventricular function. The model was shown to be capable of predicting changes in pump variables following changes in the state of the heart, possibly aiding pump controller design in future. The fluid-electromechanics model enables study of the link between electrical activation, myocardial mechanics and blood hemodynamics characteristics, allowing holistic simulations of new therapeutic approaches in their ideation stage, aiding to speed up technology transfer to pre-clinical and clinical trial stages.

## Author contributions

AAB carried out the computational modeling. AAB, AA, and SD developed the framework for the standard model. AAB and AA drafted the manuscript. AAB, AA, MS, NL, and SD developed the framework for LVAD simulations, conceived of and coordinated the study, reviewed the manuscript and gave final approval for publication.

### Conflict of interest statement

The authors declare that the research was conducted in the absence of any commercial or financial relationships that could be construed as a potential conflict of interest.
