# Supplementary material for: A Multiphysics Biventricular Cardiac Model: Simulations With a Left-Ventricular Assist Device
Source: Front Physiol. 2018 Sep 11;9:1259. doi: 10.3389/fphys.2018.01259 (PMC6142745; doi:10.3389/fphys.2018.01259)
Supplement: Supplementary file 6 [file Data_Sheet_1.pdf]

Supplementary Material: A Multiphysics Biventricular Cardiac Model: Simulations with a Left-Ventricular Assist Device

Azam Ahmad Bakir [1,*], Amr Al Abed [1], Michael Stevens [1,2] Nigel H. Lovell [1] and Socrates Dokos [1]

[1]*Graduate School of Biomedical Engineering, University of New South Wales, Kensington, NSW 2052, Australia,* [2]*Innovative Cardiovascular Engineering and Technology Laboratory, Critical Care Research Group, The Prince Charles Hospital, Brisbane, QLD*

Correspondence*: Azam Ahmad Bakir (a.ahmadbakir@unsw.edu.au)

1 MICROSTRUCTURE

Based on the wall distance from each of the boundaries (epicardium and endocardium), a local fiber angle was assigned within the myocardium domain. Using the in-built wall distance tool in COMSOL, the distance of each position in the domain with respect to an origin boundary wall was determined. COMSOL's wall distance tool utilized the method given by Fares and Schrder (2002), based on modified Eikonal equations as in Eqs. (S1) and (S2).

$$\nabla G \cdot \nabla G + \sigma_w G(\nabla \cdot \nabla G) = (1 + 2\sigma_w)G^4 \tag{S1}$$

$$D = \frac{1}{G} - \frac{2}{l_{ref}} \tag{S2}$$

where G is the reciprocal of distance D to the origin wall. D was calculated for both endocardium and epicardium, D_{endo}, and D_{epi}. D_{endo} was calculated with the endocardium set as the origin wall, whilst D_{epi} was calculated with the epicardium set as the origin wall. The smoothing parameter, σ_w was set to 0.2. At the origin wall, G was set to be $\frac{2}{l_{ref}}$, where l_{ref} was set to 0.07 cm. This setting gives us a sufficiently accurate measure of distance to the origin wall for distances greater than l_{ref}, or 0.07 cm.

A transmural index, γ, was defined equal to 1 at the epicardium and 0 at the endocardium. The difference of the two wall distance variables, $d = D_{endo} - D_{epi}$, produced a linear relation with respect to the wall distance. Variable γ in Eq. (S3) was calculated using $\overline{d_{endo}}$ and $\overline{d_{epi}}$, which are the average of d at the endocardial and epicardial surfaces respectively.

$$\gamma = \frac{d - \overline{d_{endo}}}{\overline{d_{epi}} - \overline{d_{endo}}} \tag{S3}$$

By using γ, the fiber angle, α_f, can be assigned within the myocardium in accordance with Eq (S4).

$$\alpha_f = (1 - \gamma)\alpha_{f,endo} - \gamma\alpha_{f,epi} \tag{S4}$$

where $\alpha_{f,epi}$ is -60 ° and $\alpha_{f,endo}$ is 60 °. The fiber and sheet orientations were defined based on the local boundary tangent bases vectors, $(\vec{e_1}, \vec{e_2}, \vec{e_3})$ shown in Fig. S1, similar to the approach of Eriksson et al. (2013). Tangent bases vectors of the ellipsoid were organized such that the first basis vector, $\vec{e_1}$, was aligned along the long-axis direction of the ellipsoid and the second basis, $\vec{e_2}$, aligned along the circumferential direction, as shown in Fig. S1. Normal to $\vec{e_1}$ and $\vec{e_2}$ is the third basis vector, $\vec{e_3}$. By default in COMSOL, these tangent basis vectors are defined only at the boundary. To define the base vectors within the myocardial wall, these local boundary tangent vectors at the endocardium and epicardium were extruded into the myocardium by using COMSOL's general extrusion tool. In brief, all points with the same values of $\frac{X}{r}$, $\frac{Y}{r}$ and $\frac{Z}{r}$ were assigned the same local boundary tangent vectors. X, Y, Z are the material coordinates of the points within the domain, whilst r is $\sqrt{X^2 + Y^2 + Z^2}$. The final myocardial basis unit vectors were determined by weighting the boundary basis vectors at the endocardium and epicardium as shown in Eq. (S5) using the transmural index γ variable, defined earlier.

$$(\hat{e_1}, \hat{e_2}, \hat{e_3})\big|_{myo} = (1 - \gamma)(\hat{e_1}, \hat{e_2}, \hat{e_3})\big|_{endo} + \gamma(\hat{e_1}, \hat{e_2}, \hat{e_3})\big|_{epi} \tag{S5}$$

The fiber vector, $\vec{F}$, and the normal-to-sheet vector, $\vec{N}$, were assumed to lie within the plane of $\vec{e_1}$ and $\vec{e_2}$. The fiber vector orientation was set to -60° from $\vec{e_2}$ in the epicardium and 60° in the endocardium, while the transition between these angles in the myocardium was based on α_f. The normal-to-sheet, $\vec{N}$, was assumed to be oriented orthogonal to the fiber vector, $\vec{F}$. The sheet vector, $\vec{S}$ was assumed to be perpendicular to the endocardium and epicardium; thus, it lies along the $\vec{e_3}$ direction.

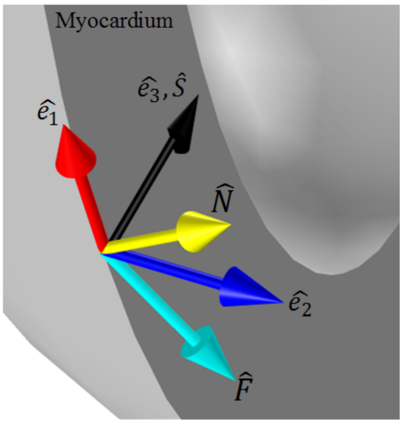

Figure S1. Local boundary tangent vectors $(\hat{e_1}, \hat{e_2}, \hat{e_3})$, illustrated on the epicardial and surface of the left ventricle. The first and second basis vectors, $\hat{e_1}$ and $\hat{e_2}$, are aligned tangential to the surface such that $\hat{e_1}$ lies in the vertical plane through the ventricular major axis and $\hat{e_2}$ lies in the circumferential plane parallel to the ventricular base. The third basis vector, $\hat{e_3}$, is normal to the surface. The local fiber direction, $\hat{F}$, is oriented within the $(\hat{e_1}, \hat{e_2})$ plane at an angle α from $\hat{e_2}$. The sheet direction, $\hat{S}$, is oriented along $\hat{e_3}$. As a consequence, the normal-to-sheet vector, $\hat{N}$, also lies within the $(\hat{e_1}, \hat{e_2})$ plane.

1.1 Special Consideration for the Right Ventricle

Since the two ventricles differ in thickness, the microstructure definitions were computed separately for each ventricle, with values at the interventricular sulci subsequently linearly interpolated.

Since the geometry was built from ellipsoids, an ellipsoid equation was used to define the LV and RV regions, using the variable η in Eq. (S6), where R_{LV} is the LV outer radius along the X and the Y material coordinate axes and $R_{Z,LV}$ is the LV outer radius along the Z-coordinate axis. The ellipsoid variable η has a value of less than one within the LV ellipsoid, with the RV was defined as regions with η more than 1 using the variable η' in Eq. (S7) and in Fig. S2. A step function was employed to smoothen the transition of η' from LV to RV, with a transition size of 0.3.

$$\eta = (\frac{X}{R_{LV}})^2 + (\frac{Y}{R_{LV}})^2 + (\frac{Z}{R_{Z,LV}})^2 \tag{S6}$$

$$\eta' = \begin{cases} 1 & \eta \leq 1, leftventricle \\ 1 - \left(0.5 + 0.75\left(\frac{\eta}{h}\right) - 0.25\left(\frac{\eta}{h}\right)^3\right) & 1 < \eta \leq 1.3, transitionzone \\ 0 & \eta > 1.3, rightventricle \end{cases} \tag{S7}$$

Similar to the LV, the fiber orientation was set to 60° at the endocardium and -60° at the epicardium. The sheet direction was simply assumed to be perpendicular to the endocardial and epicardial surfaces, interpolated within the myocardium as described previously. The septal surface within the RV was defined as an epicardial boundary of the LV similar to the definition made by Sermesant et al. (2005).

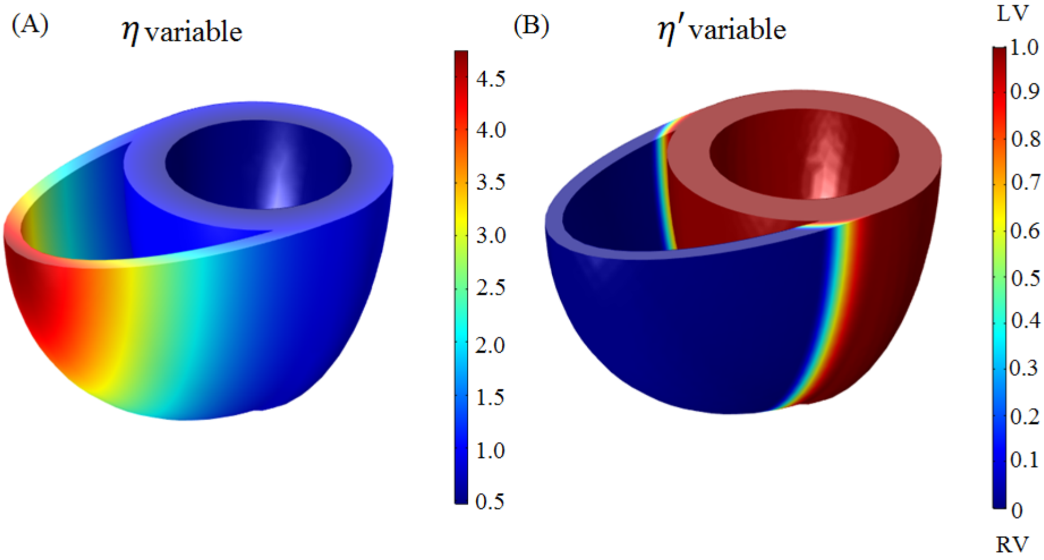

Figure S2. (A) η variable defined by Eq. (S6). Using η' variable, the LV region is defined to be 1 and the RV to be zero. Transition between LV and RV regions was handled by a smoothed step function with transition size was set to 0.3 to ensure smooth transition.

The transmural index, γ and local fiber vector definition were computed separately for the LV and RV, whilst the transition between both was handled according to Eq. (S7). To define the local basis vectors in the RV, the basis vectors at the epicardium and endocardium were extruded radially such that points with the same $\frac{X}{r}$, $\frac{Y}{r}$ and $\frac{Z}{r}$ values were assigned the same local basis vectors. The resulting microstructure obtained is shown in Fig. S3.

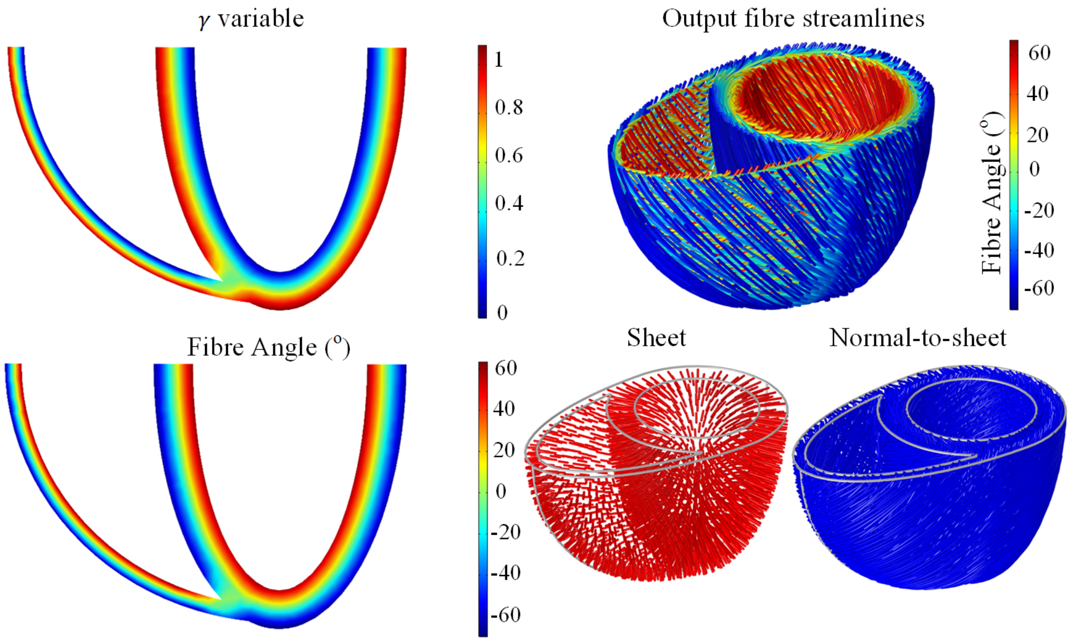

Figure S3. Microstructure in the biventricular model. (A) Cross-sectional view of the output transmural index γ, (B) output fiber angle, overlaid on streamlines representing the fibers, and (C) microstructure sheet, and normal-to-sheet orientations.

2 LIST OF PARAMETERS AND INITIAL VALUES

In this section, the parameters used in the model are listed. Table S1 listed all the parameters used for electrical activation model (Eqs. (1) - (6) of the main text). The active stress formulation parameters for Eqs. (7) and (8) of the main text are described in Table S2 below. The parameters for our model were based on the cited literatures or manually tuned as described below.

Table S1. Parameter and initial variable values for electrophysiology formulations. Several parameters do not have physiological meaning as the formulations are merely phenomenological-type. Descriptions of the parameters are available in the original paper (Nash and Panfilov, 2004) unless mentioned below.

Parameter	Values	Description
β_{sv}	$160000\, m^{-1}$	Membrane surface to volume ratio. β_{sv} can be approximated from the cell's radius, r_{cell} from the relation $\beta_{sv} = 2/r_{cell}$, where r_{cell} was assumed to be 25 μm (Tracy and Sander, 2011).
C_m	$0.01\ F\, m^{-2}$	Membrane capacitance
σ_f	$2\ S\, m^{-1}$	Electrical conductivity along $\hat{F}$ direction. This is set to ensure that myocardial activation is within the limit of normal QRS duration (80-120 ms) (Guyton and Hall, 2006).
k_1	8	Based on Nash and Panfilov (2004)
k_2	$1\ F\, m^{-2} s^{-1}$	Based on Nash and Panfilov (2004) for myocardium
	$2\ F\, m^{-2} s^{-1}$	For Purkinje fiber. Adjusted to achieve twice myocardial upstroke velocity
A	$0.1\ V$	Based on Nash and Panfilov (2004)
B	$-0.08\ V$	Based on Nash and Panfilov (2004)
a	0.12	At epicardium
	0.07	At endocardium
	0.095	At Purkinje fiber
		*To obtain a realistic recovery sequence, where the epicardium relaxed earlier than the endocardium, parameter a was modulated such that action potential duration was longest in the endocardium and shortest in the epicardium (Glukhov et al., 2010).
ε_0	$0.2\ s^{-1}$	Based on Nash and Panfilov (2004)
μ_1	$20\ s^{-1}$	Based on Nash and Panfilov (2004)
μ_2	0.3	Based on Nash and Panfilov (2004) for myocardium
	0.7	For Purkinje fiber. Parameter is adjusted to increase the action potential duration by 25%
ρ_i	$33\ \Omega\ cm$	Purkinje resistivity. Parameter is set to achieve approximately three-fold increase in conduction velocity
Initial Values		
V	$-0.08\ V$	Membrane potential
R	0.02	Recovery variable

All parameters for Eqs. (7) and (8), shown in Table S2, were manually adjusted, so that the active stress duration matched cardiac muscle isometric twitch duration reported in isolated human cardiomyocytes (Hasenfuss et al., 1992). Parameter ϵ_0 controls the rate of active stress relaxation, with higher ϵ_0 resulting in a faster relaxation. The time to peak active stress was mainly controlled by parameter ϵ_∞, and generally increased with an increase in ϵ_∞. However, it should be noted that by altering parameter ϵ_∞, the total active stress magnitude will also be affected. This can be compensated for by simply changing parameter k_{Ta} to yield the desirable deformation level. Parameter ξ affects mainly the relaxation rate and time to peak, with a higher value of ξ leading to a reduction relaxation rate and a very small increase in activation

rate. Parameter k_{Ta}, which represents maximum active stress magnitude, was adjusted later on to obtain desirable stroke volume.

Table S2. Parameter and initial variable values for the active stress formulations. Several parameters do not have physiological meaning as the formulations are merely phenomenological-type.

Parameter	Values
ϵ_0	28 s^{-1}
ϵ_∞	5.7 s^{-1}
ξ	7.6 V^{-1}
A	0.1 V
B	$-0.08\ V$
$V_{threshold}$	-0.03 V
k_{Ta}	135 kPa
Initial Values	
T_a	$0\ kPa$

Mechanical parameters for the myocardial constitutive law (Eqs. (9) - (12) of the main text) and equation of motion (Eq. (13)) are listed in the Table S3, along with fluid mechanics parameters (Eqs. (15) - (19)).

Table S3. Parameter values and initial variables for fluid-structure interactions. Parameters are based on (Holzapfel and Ogden, 2009; Watanabe et al., 2004) while parameters for moving mesh (C_1, C_2, and κ_{mesh}) are set by default in COMSOL.

Parameters	Value	Description
κ	250 kPa	Bulk modulus
a_i	2.280 kPa	Isotropic material property
b_i	9.726	Isotropic material property
a_f	1.685 kPa	Fibre direction material property
b_f	15.779	Fibre direction material property
ρ_s	1370 $kg\ m^{-3}$	Myocardial density
α	100 s^{-1}	Rayleigh damping parameter
β	0.01 s	Rayleigh damping parameter
μ_f	0.0035$Pa\ s$	Blood viscosity
ρ_f	1060 kgm^{-3}	Blood density
C_1	1	Artifical shear moduli parameter for moving mesh
C_2	0	Non-linear mesh stiffening parameter for moving mesh
	100	For LVAD model
κ_{mesh}	1	Artifical bulk moduli parameter for moving mesh
Initial values		
$\mathbf{u_s}$	$\mathbf{0}$ cm	
$\mathbf{v_f}$	$\mathbf{0}$ m s^{-1}	
p	0 mmHg	

The parameters for the left ventricular assist device (LVAD) model in Eqs.(24) to (28) of the main text were adopted from the Lim et al. (2010) paper. The material properties of the silicone cannula were selected from COMSOL library. These parameters are listed and described in Table S4.

Table S4. List of parameters used in the LVAD model based on Lim et al. (2010).

Parameter	Values	Descriptions
k_e	8.48x10^{-3} V s rad^{-1}	Back electromotive force (BEMF) constant
R_{pump}	1.38 Ω	Motor windings resistance
L	0.027 mmHg s^2 ml^{-1}	Inductance induced by the blood inertia
k_{pump}	1 V s rad^{-1}	LVAD proportional controller constant
J	7.74x10^{-6} kg m^2	Moment of inertia of the pump impeller
a_p	1576.8 kg s m^{-4} rad^{-1}	Pump constant obtained under empirical fitting
b_p	7.14x10^{-4} kg s m^{-1} rad^{-2}	Pump constant obtained under empirical fitting
c_p	1.92x10^{-5} kg m^2 s^{-1} rad^{-1}	Pump constant obtained under empirical fitting
d_p	3.14x10^{-10} kg m^2 s^{-1} rad^{-3}	Pump constant obtained under empirical fitting
e_p	-6 mmHg	Pump constant obtained under empirical fitting
f_p	-0.0524 mmHg min^3 l^{-3}	Pump constant obtained under empirical fitting
g_p	0.0019 mmHg s^2 rad^{-2}	Pump constant obtained under empirical fitting
k_r	0.006 mmHg s^2 ml^{-2}	Proportionality constant for flow-dependent resistance
E	170 GPa	Silicone cannula's Young's modulus
ν	0.28	Silicone cannula's Poisson's ratio
ρ_s	2329 kg m^{-3}	Silicone cannula's density

3 WINDKESSEL CIRCULATION

The Windkessel variables and parameters are abbreviated as follows:

1. V = compartmental volume
2. Q = compartmental flow rate
3. R = flow resistance
4. C = compartmental capacitance
5. P = pressure

The following abbreviations of the subscript indicate the Windkessel compartment of the variables' and parameters':

1. as = systemic artery
2. vs = systemic venous
3. ap = pulmonary artery
4. vp = pulmonary venous
5. la = left atrium
6. ra = right atrium
7. ao = aortic valve

8. pa = pulmonic valve
9. tri = tricuspid valve
10. mi = mitral valve
11. lv = left ventricle
12. rv = right ventricle

The rates of volumetric change for each circulatory compartments were calculated using the list of Eqs. (S8) below. In general, the rate of volumetric change can be obtained by the difference between inflow and outflow rates of each compartment. The pressure for each compartment can then be obtained by the linear relation with the volume listed in Table S5. Each flow rate, Q, can be determined using Eq. (S9).

$$
\begin{aligned}
\frac{\partial V_{as}}{\partial t} &= Q_{ao} - Q_{as} \\
\frac{\partial V_{vs}}{\partial t} &= Q_{as} - Q_{vs} \\
\frac{\partial V_{ra}}{\partial t} &= Q_{vs} - Q_{tri} \\
\frac{\partial V_{ap}}{\partial t} &= Q_{pa} - Q_{ap} \\
\frac{\partial V_{vp}}{\partial t} &= Q_{ap} - Q_{vp} \\
\frac{\partial V_{la}}{\partial t} &= Q_{vp} - Q_{mi}
\end{aligned} \tag{S8}
$$

$$
\begin{aligned}
Q_{ao} &= \frac{P_{lv,aortic} - P_{as}}{R_{ao}} \\
Q_{as} &= \frac{P_{as} - P_{vs}}{R_{as}} \\
Q_{vs} &= \frac{P_{vs} - P_{ra}}{R_{vs}} \\
Q_{tri} &= \frac{P_{ra} - P_{rv,tri}}{R_{tri}} \\
Q_{pa} &= \frac{P_{rv,pa} - P_{ap}}{R_{pa}} \\
Q_{ap} &= \frac{P_{ap} - P_{vp}}{R_{ap}} \\
Q_{vp} &= \frac{P_{vp} - P_{la}}{R_{vp}} \\
Q_{mi} &= \frac{P_{la} - P_{lv,mi}}{R_{mi}}
\end{aligned} \tag{S9}
$$

The aortic (Q_{ao}), mitral (Q_{mi}), pulmonary artery (Q_{pa}) and tricuspid (Q_{tri}) flow rates determined by Eq. (S9) were prescribed to the inlet and outlet boundaries of the finite element model, with P_{lv} and P_{rv} obtained from the finite element model at the respective surfaces. Other pressure variables in Eq.

(S9) were obtained from the linear relation with the Windkessel capacitances described in Table S5. The resistances and unstressed volumes are listed in Tables S6 and S7, respectively.

In brief, the resistance and capacitance parameters were determined based on the mean circulatory pressure and blood volume distribution in healthy human circulation described by Guyton and Hall (2006). Assuming the Windkessel circuit is at steady state with the left ventricle supplying 5 L min^{-1} of blood, the resistances were calculated using the relation in Eqs. (S9). Then, assuming the right ventricle as passive capacitance at constant 120 ml and total blood volume as 5 L, the capacitances were optimized using MATLAB (Mathworks, MA, USA) in-built "fminsearch" tool until the mean blood volumes distribution was obtained. The final initial values of Windkessel volumes for Eqs. (S8) are listed in Table S8, which were obtained after running multiple cardiac cycles and extrapolated to steady state.

Table S5. Circulatory capacitance parameter in human. Baseline left and right atrial pressures, $P_{la,baseline}$ and $P_{ra,baseline}$, were set to 5 mmHg each.

Parameter	Equation	Value (ml/mmHg)
Systemic arterial capacitance, C_{as}	$C_{as} = \frac{V_{as}-V_{as,unstress}}{P_{as}}$	2.52
Systemic venous capacitance, C_{vs}	$C_{vs} = \frac{V_{vs}-V_{vs,unstress}}{P_{vs}}$	58.6
Right atrial capacitance, C_{ra}	$C_{ra} = \frac{V_{ra}-V_{ra,minimum}}{P_{ra}-P_{ra,baseline}}$	19.4
Pulmonary arterial capacitance, C_{ap}	$C_{ap} = \frac{V_{ap}-V_{ap,unstress}}{P_{ap}}$	8.01
Pulmonary venous capacitance, C_{vp}	$C_{vp} = \frac{V_{vp}-V_{vp,unstress}}{P_{vp}}$	12.7
Left atrial capacitance, C_{la}	$C_{la} = \frac{V_{la}-V_{la,minimum}}{P_{la}-P_{la,baseline}}$	4.89

Table S6. Unstressed compartmental volumes in normal human (mean ± standard deviation)

Quantity	Source	Measured at	Values (ml)
Systemic Arterial Volume, V_{as}	Guyton and Hall (2006)	Unstressed	400
Systemic Venous Volume, V_{vs}	Lim et al. (2010)	Unstressed	2112.27
Pulmonary Arterial Volume, V_{ap}	Lim et al. (2010)	Unstressed	91.67
Pulmonary Venous Volume, V_{vp}	Lim et al. (2010)	Unstressed	132.39
Left Atrial Volume, V_{la}	Hudsmith et al. (2005)	Minimum	44±13
Right Atrial Volume, V_{ra}	Järvinen et al. (1994)	Minimum	72±18

Table S7. Circulatory resistance parameters in human

Parameter	Value (mmHg s ml^{-1})
Aortic resistance, R_{ao}	0.042
Systemic arterial resistance, R_{as}	0.84
Systemic venous resistance, R_{vs}	0.22
Tricuspid valve resistance, R_{tri}	0.0042
Pulmonary valve resistance, R_{pa}	0.025
Pulmonary arterial resistance, R_{ap}	0.024
Pulmonary venous resistance, R_{vp}	0.012
Mitral valve resistance, R_{mi}	0.0054

Table S8. Stable end-diastole Windkessel compartment initial volumes

Volume compartment	Symbol	Values (ml)
Left ventricle	$V_{lv,\infty}$	148.6
Right ventricle	$V_{rv,\infty}$	143.5
Systemic artery	$V_{as,\infty}$	591.2
Systemic venous	$V_{vs,\infty}$	3457.4
Pulmonary artery	$V_{ap,\infty}$	200.4
Pulmonary venous	$V_{vp,\infty}$	298.5
Left atrium	$V_{la,\infty}$	83.2
Right atrium	$V_{ra,\infty}$	77.2

4 MESH MEASURES

Table S9. Mesh size for the standard model. Mesh element size was calculated as the length of the longest edges in the element.

Mesh setting	Normal	Fine	Dilated heart LVAD
Average element size in myocardium (cm)	0.57	0.42	0.52
Average element size in blood domain (cm)	0.49	0.37	0.41
Maximum element size in myocardium (cm)	0.92	0.61	0.74
Maximum element size in blood domain (cm)	0.76	0.61	0.64
Minimum element size in myocardium (cm)	0.16	0.10	0.14
Minimum element size in blood domain (cm)	0.08	0.01	0.03
Number of boundary layer mesh in blood domain	2	2	2
Average inlet/outlet edges element size (cm)	0.1	0.1	0.1
Number of elements in myocardium (cm)	22661	51249	28005
Number of elements in blood domain (cm)	44078	83321	103569

REFERENCES

Eriksson, T., Prassl, A., Plank, G., and Holzapfel, G. (2013). Influence of myocardial fiber/sheet orientations on left ventricular mechanical contraction. *Mathematics and Mechanics of Solids* doi:10.1177/1081286513485779

Fares, E. and Schrder, W. (2002). A differential equation for approximate wall distance. *International Journal for Numerical Methods in Fluids* 39, 743–762. doi:10.1002/d.348

Glukhov, A. V., Fedorov, V. V., Lou, Q., Ravikumar, V. K., Kalish, P. W., Schuessler, R. B., et al. (2010). Transmural dispersion of repolarization in failing and nonfailing human ventricle. *Circulation Research* 106, 981–991. doi:10.1161/circresaha.109.204891

Guyton, A. and Hall, J. (2006). *Textbook of medical physiology* (Elsevier Saunders)

Hasenfuss, G., Mulieri, L. A., Leavitt, B. J., Allen, P. D., Haeberle, J. R., and Alpert, N. R. (1992). Alteration of contractile function and excitation-contraction coupling in dilated cardiomyopathy. *Circulation Research* 70, 1225–32. doi:10.1161/01.res.70.6.1225

Holzapfel, G. A. and Ogden, R. W. (2009). Constitutive modelling of passive myocardium: a structurally based framework for material characterization. *Philosophical Transactions of the Royal Society A: Mathematical, Physical and Engineering Sciences* 367, 3445–3475. doi:10.1098/rsta.2009.0091

Hudsmith, L. E., Petersen, S. E., Francis, J. M., Robson, M. D., and Neubauer, S. (2005). Normal human left and right ventricular and left atrial dimensions using steady state free precession magnetic resonance imaging. *Journal of Cardiovascular Magnetic Resonance* 7, 775–782. doi: 10.1080/10976640500295516

Järvinen, V. M., Kupari, M. M., Hekali, P. E., and Poutanen, V. P. (1994). Right atrial MR imaging studies of cadaveric atrial casts and comparison with right and left atrial volumes and function in healthy subjects. *Radiology* 191, 137–142. doi:10.1148/radiology.191.1.8134560

Lim, E., Dokos, S., Cloherty, S. L., Salamonsen, R. F., Mason, D. G., Reizes, J. A., et al. (2010). Parameter-optimized model of cardiovascular:rotary blood pump interactions. *IEEE Transactions on Biomedical Engineering* 57, 254–266. doi:10.1109/TBME.2009.2031629

Nash, M. P. and Panfilov, A. V. (2004). Electromechanical model of excitable tissue to study reentrant cardiac arrhythmias. *Progress in Biophysics and Molecular Biology* 85, 501–522. doi: 10.1016/j.pbiomolbio.2004.01.016

Sermesant, M., Rhode, K., Sanchez-Ortiz, G. I., Camara, O., Andriantsimiavona, R., Hegde, S., et al. (2005). Simulation of cardiac pathologies using an electromechanical biventricular model and XMR interventional imaging. *Medical Image Analysis* 9, 467–480. doi:10.1016/j.media.2005.05.003

Tracy, R. E. and Sander, G. E. (2011). Histologically measured cardiomyocyte hypertrophy correlates with body height as strongly as with body mass index. *Cardiology Research and Practice* 2011. doi: 10.4061/2011/658958

Watanabe, H., Sugiura, S., Kafuku, H., and Hisada, T. (2004). Multiphysics simulation of left ventricular filling dynamics using fluid-structure interaction finite element method. *Biophysical Journal* 87, 2074–2085. doi:10.1529/biophysj.103.035840
